# Supplementary material for: Recruitment of autophagy initiator TAX1BP1 advances aggrephagy from cargo collection to sequestration
Source: EMBO J. 2024 Oct 24;43(23):9. doi: 10.1038/s44318-024-00280-5 (PMC11611905; doi:10.1038/s44318-024-00280-5)
Supplement: Supplementary file 1 — Appendix [file 44318_2024_280_MOESM1_ESM.pdf]

Appendix:

Recruitment of autophagy initiator TAX1BP1 advances aggrephagy from cargo collection to sequestration

Table of Contents

Appendix Figure S1…………………………………………………………………..page 2

Appendix Figure S2…………………………………………………………………..page 3

Appendix Figure S1

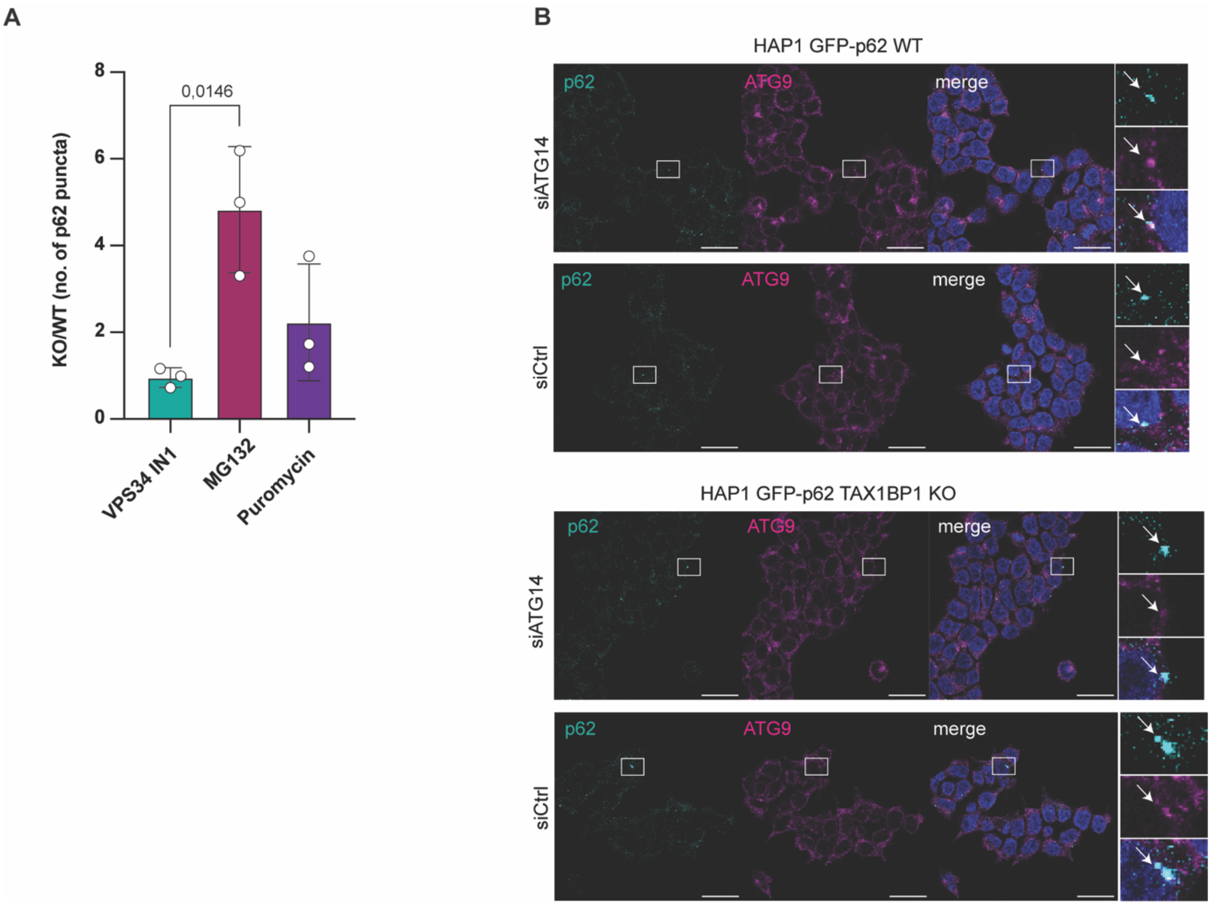

Appendix Figure S1: TAX1BP1 is crucial for autophagic flux of p62 condensates in cells. A) Quantification of the relative increase in the number of p62 puncta between the indicated treatments. B) Representative immunofluorescence images of GFP-p62 WT and TAX1BP1 KO cells stained for ATG9 after siRNA-mediated knock-down of ATG14 (scale bar = 20 μm). ATG9 colocalization with p62 puncta was abrogated in TAX1BP1 KO cell line (white arrows). Data in A shows the mean +/- s.d. from three independent experiments. One-way ANOVA with Tukey multiple comparison test was performed in B.

Appendix Figure S2

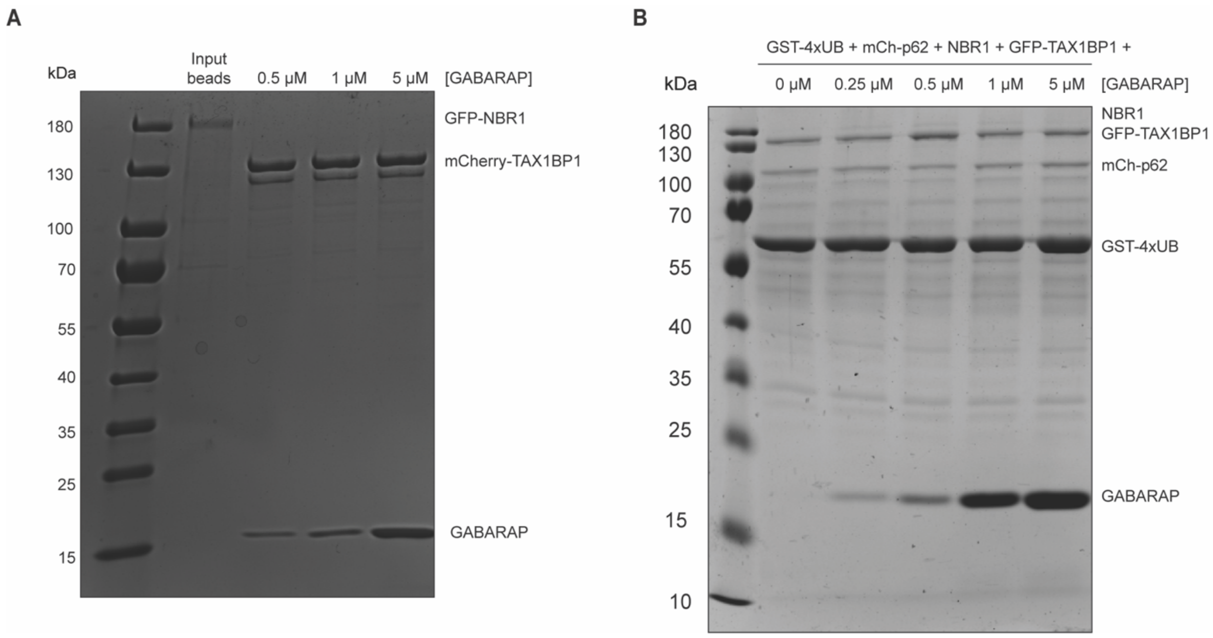

Appendix Figure S2: GABARAP competes with TAX1BP1 for NBR1 binding.

A) SDS Page gel as loading control for interaction assay in 5A. B) SDS Page gel as loading control for GABARAP competition in condensation assay in 5E.
