# Supplementary figures and images for: Recruitment of autophagy initiator TAX1BP1 advances aggrephagy from cargo collection to sequestration
Source: EMBO J. 2024 Oct 24;43(23):9. doi: 10.1038/s44318-024-00280-5 (PMC11611905; doi:10.1038/s44318-024-00280-5)

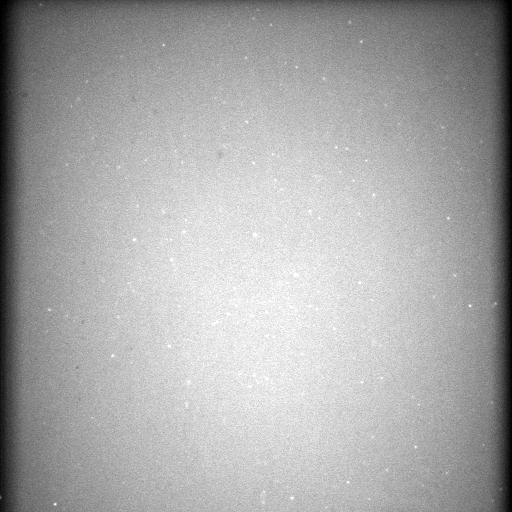

Supplement: Supplementary file 3 — Source data Fig. 1 [file 44318_2024_280_MOESM3_ESM.zip › SD figure 1/Fig1E/Condassay1_w2sd-mCherry_s8_t60.stk]

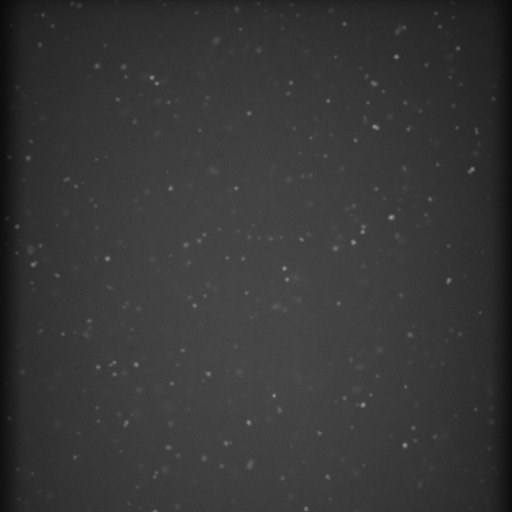

Supplement: Supplementary file 3 — Source data Fig. 1 [file 44318_2024_280_MOESM3_ESM.zip › SD figure 1/Fig1E/Condassay1_w1sd-GFP_s5_t60.stk]

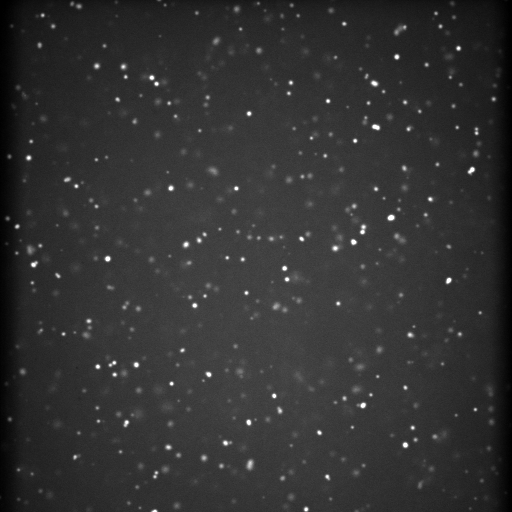

Supplement: Supplementary file 3 — Source data Fig. 1 [file 44318_2024_280_MOESM3_ESM.zip › SD figure 1/Fig1E/Condassay1_w2sd-mCherry_s5_t60.stk]

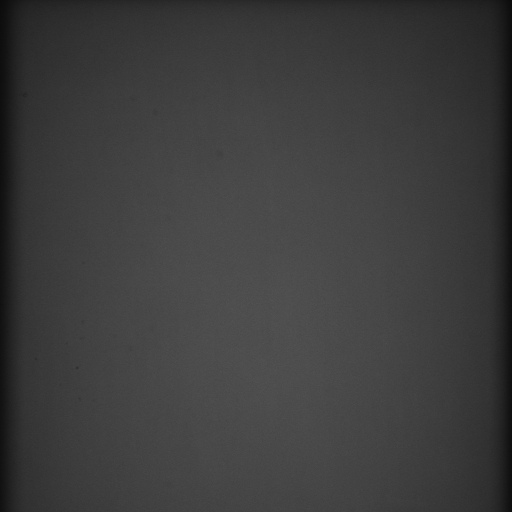

Supplement: Supplementary file 3 — Source data Fig. 1 [file 44318_2024_280_MOESM3_ESM.zip › SD figure 1/Fig1E/Condassay1_w1sd-GFP_s8_t60.stk]

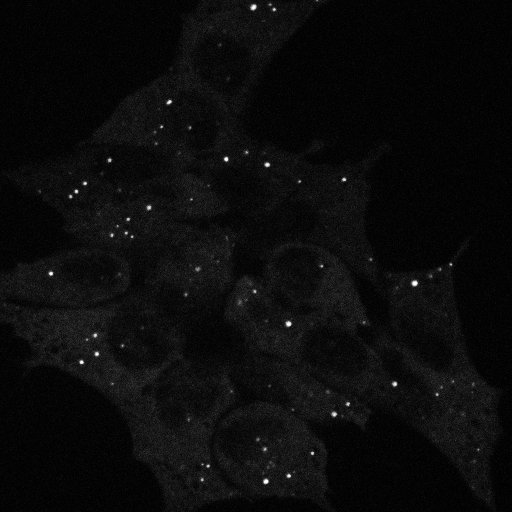

Supplement: Supplementary file 3 — Source data Fig. 1 [file 44318_2024_280_MOESM3_ESM.zip › SD figure 1/Fig1B/VPS.tif]

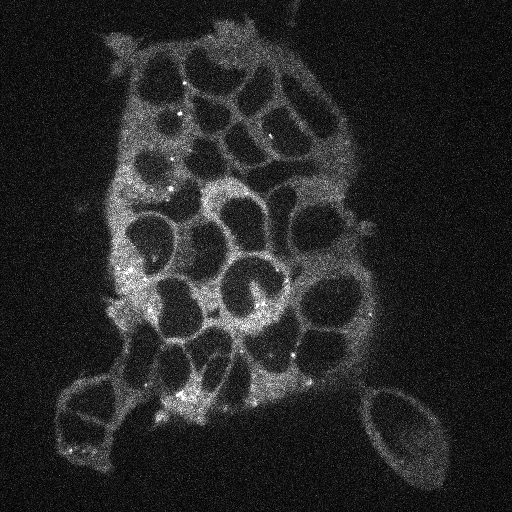

Supplement: Supplementary file 3 — Source data Fig. 1 [file 44318_2024_280_MOESM3_ESM.zip › SD figure 1/Fig1B/DMSO.tif]

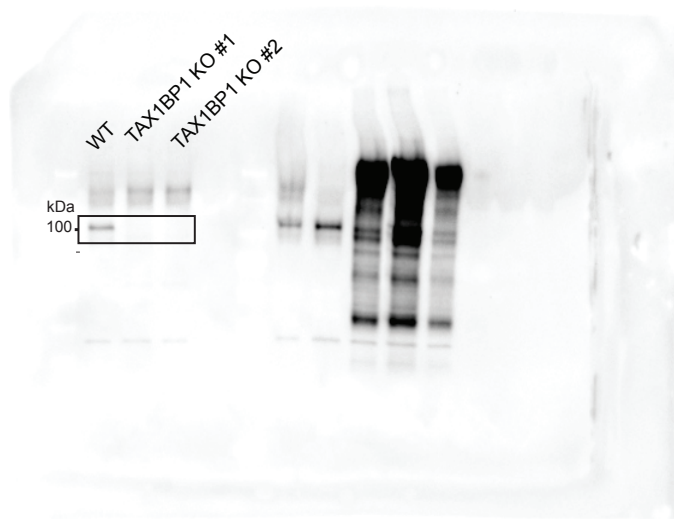

TAX1BP1 (rabbit)

(blot was 1st incubated with mouse anti NBR1

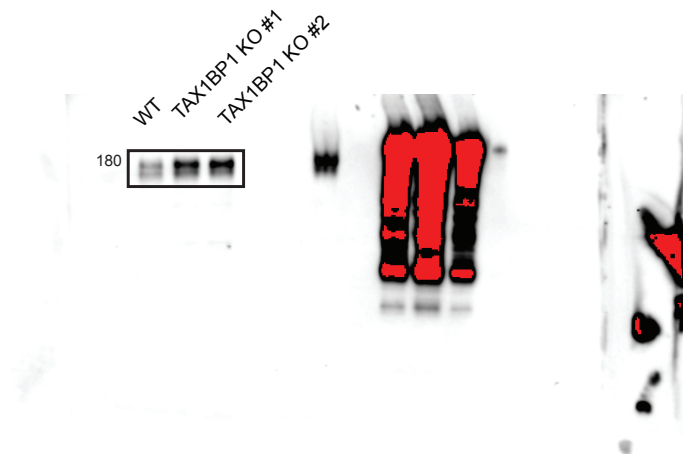

NBR1

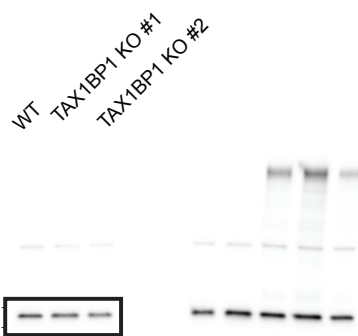

GAPDH

Supplement: Supplementary file 4 — Source data Fig. 2 [file 44318_2024_280_MOESM4_ESM.zip › SD figure 2/Fig2C.pdf]

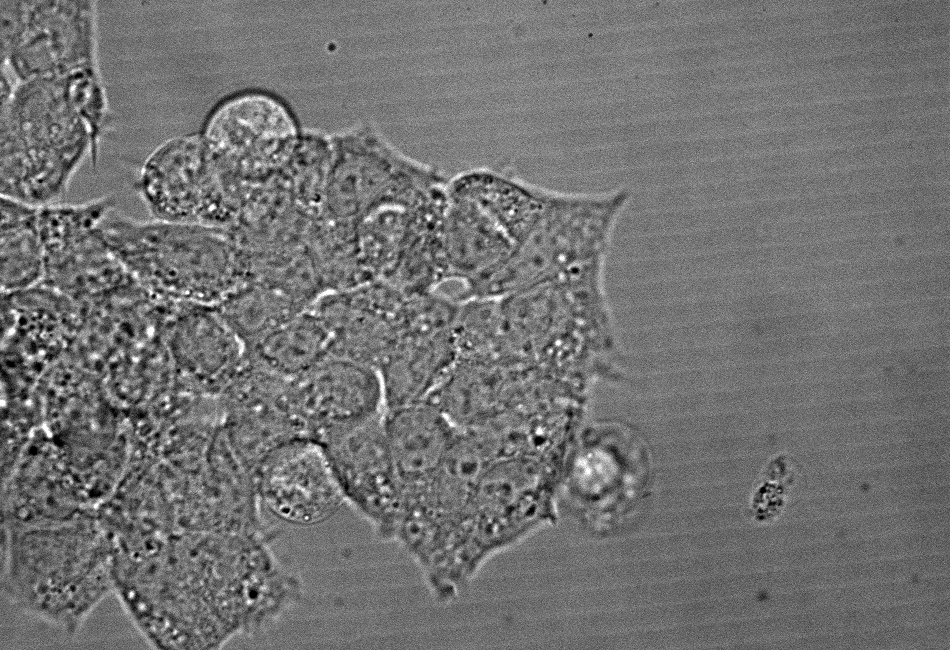

Supplement: Supplementary file 4 — Source data Fig. 2 [file 44318_2024_280_MOESM4_ESM.zip › SD figure 2/Fig2A/TAX1BP1KO_VPS.tif]

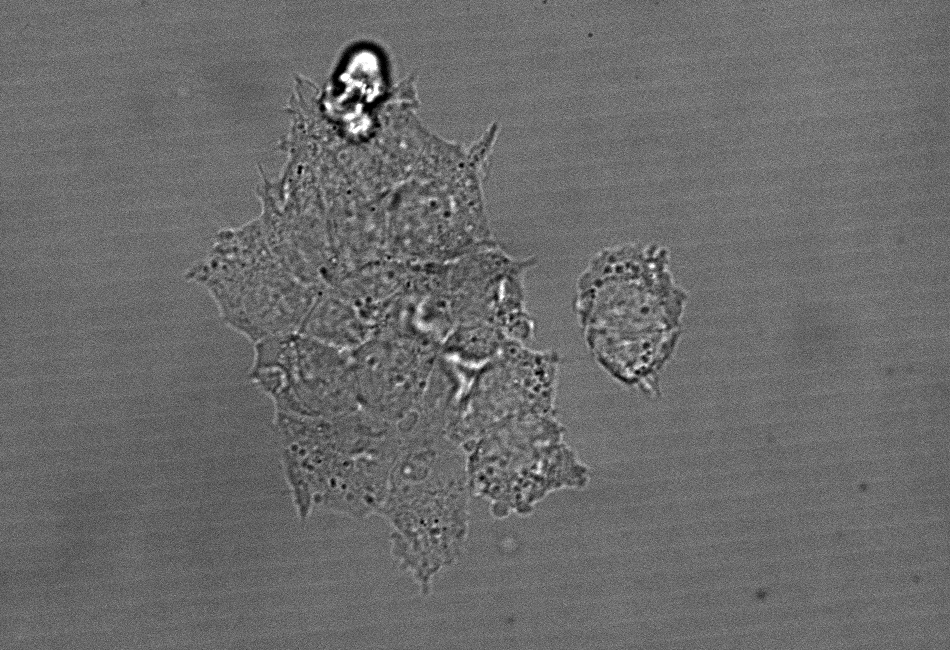

Supplement: Supplementary file 4 — Source data Fig. 2 [file 44318_2024_280_MOESM4_ESM.zip › SD figure 2/Fig2A/WT_DMSO.tif]

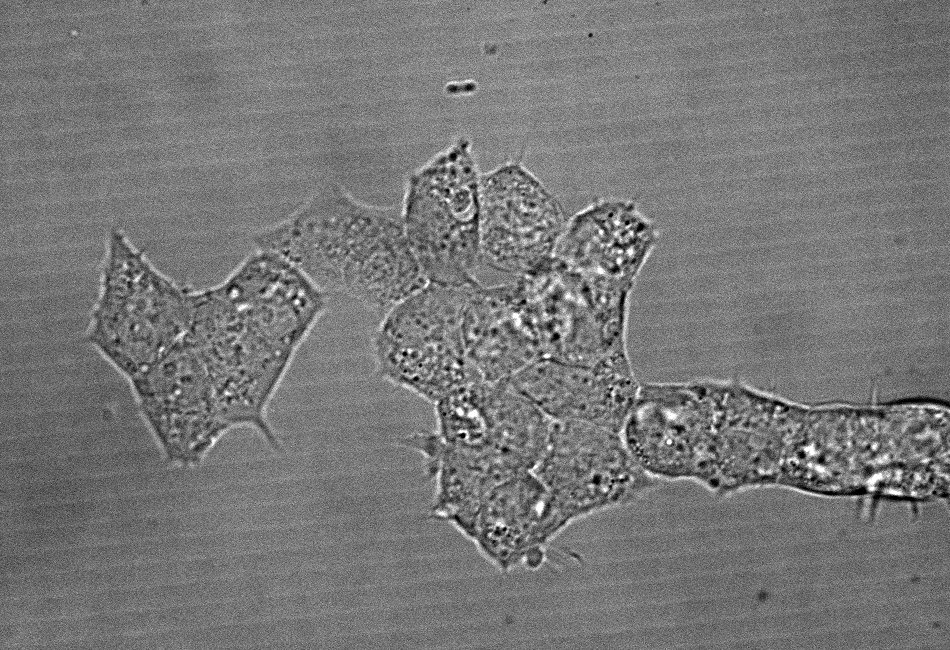

Supplement: Supplementary file 4 — Source data Fig. 2 [file 44318_2024_280_MOESM4_ESM.zip › SD figure 2/Fig2A/WT_VPS_tif.tif]

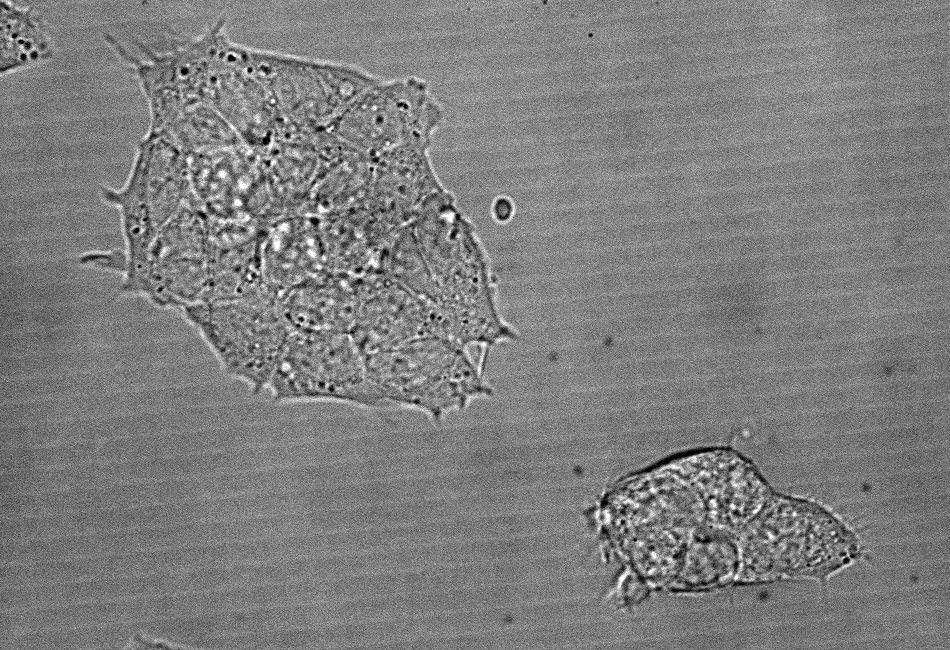

Supplement: Supplementary file 4 — Source data Fig. 2 [file 44318_2024_280_MOESM4_ESM.zip › SD figure 2/Fig2A/TAX1BP1KO_DMSO.tif]

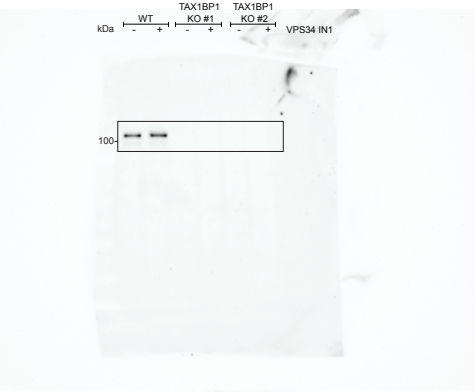

TAX1BP1

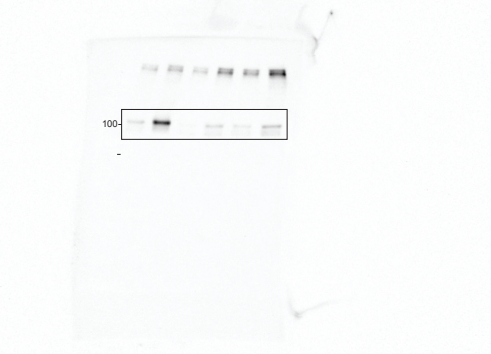

p62 p-S403

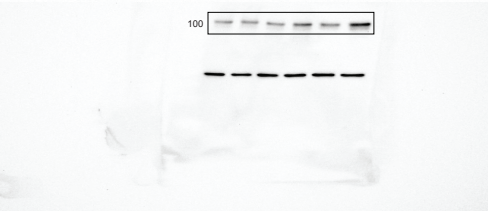

p62

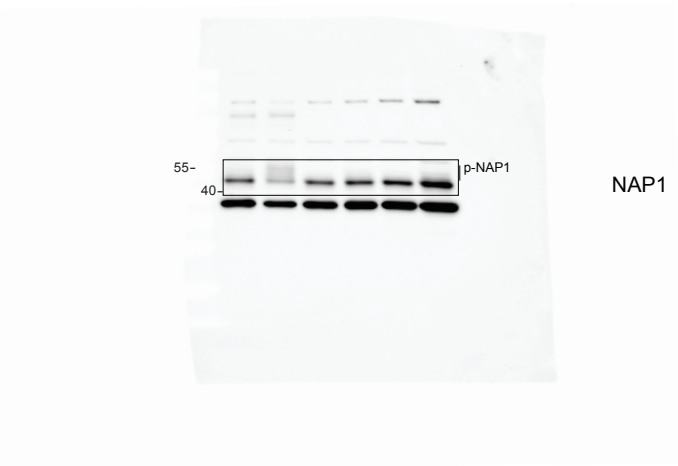

NAP1

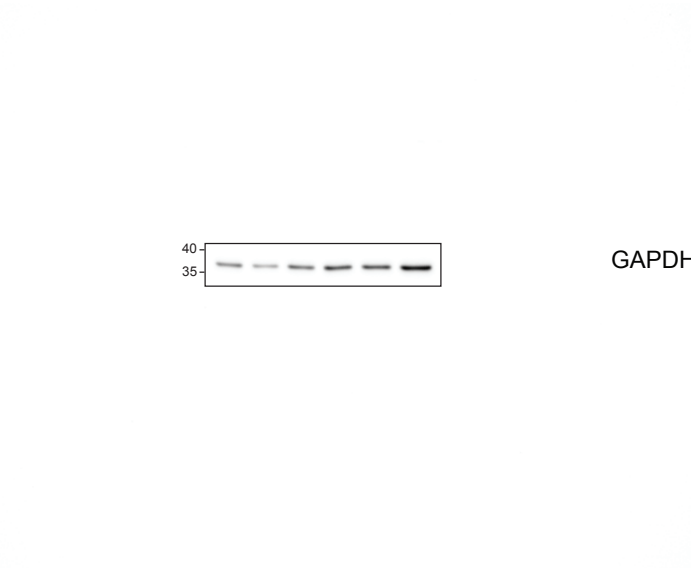

GAPDH

Supplement: Supplementary file 5 — Source data Fig. 3 [file 44318_2024_280_MOESM5_ESM.zip › SD figure 3/Fig3A.pdf]

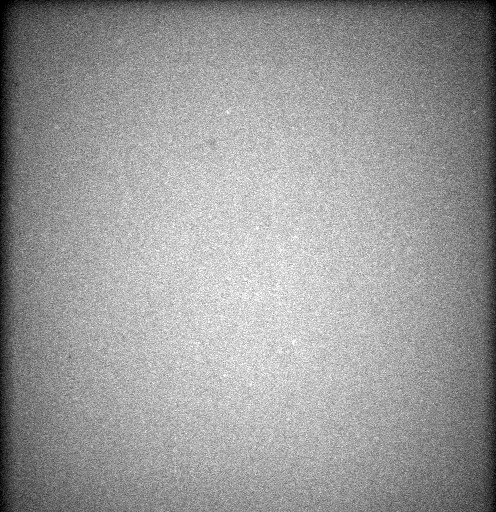

Supplement: Supplementary file 5 — Source data Fig. 3 [file 44318_2024_280_MOESM5_ESM.zip › SD figure 3/Fig3G/Condassay_GFP-Sintbad1_w1sd-GFP_s1_t30.stk]

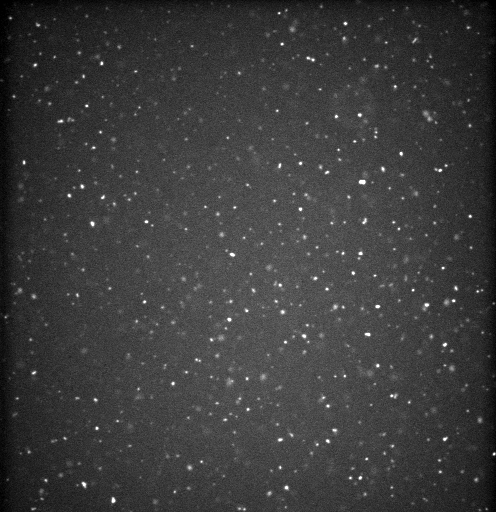

Supplement: Supplementary file 5 — Source data Fig. 3 [file 44318_2024_280_MOESM5_ESM.zip › SD figure 3/Fig3G/Condassay_GFP-Sintbad1_w2sd-mCherry_s1_t30.tif]

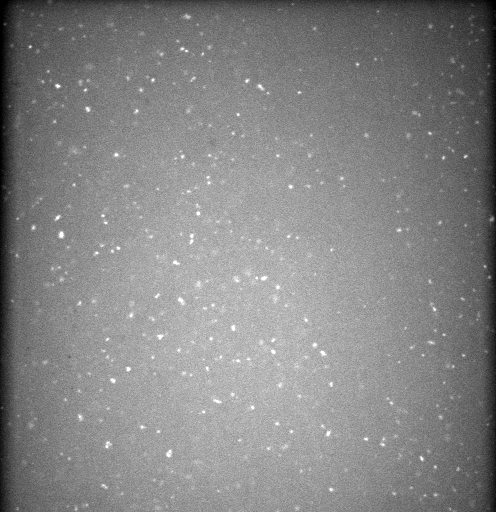

Supplement: Supplementary file 5 — Source data Fig. 3 [file 44318_2024_280_MOESM5_ESM.zip › SD figure 3/Fig3G/Condassay_GFP-Sintbad1_w1sd-GFP_s3_t30.stk]

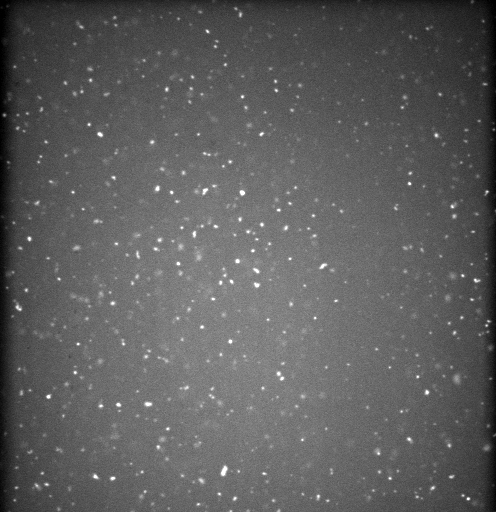

Supplement: Supplementary file 5 — Source data Fig. 3 [file 44318_2024_280_MOESM5_ESM.zip › SD figure 3/Fig3G/Condassay_GFP-Sintbad1_w2sd-mCherry_s3_t30.tif]

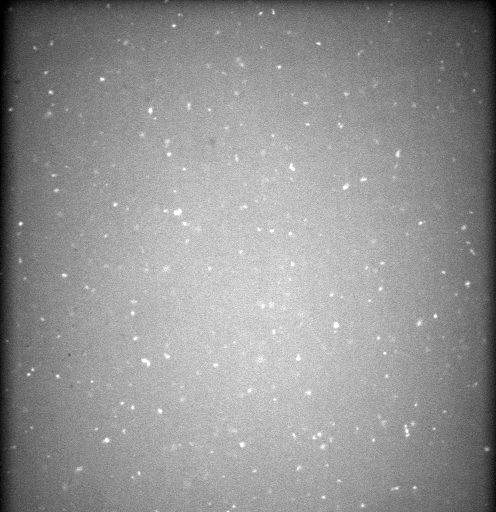

Supplement: Supplementary file 5 — Source data Fig. 3 [file 44318_2024_280_MOESM5_ESM.zip › SD figure 3/Fig3H/Condassay_TBK11_w1sd-GFP_s4_t60.stk]

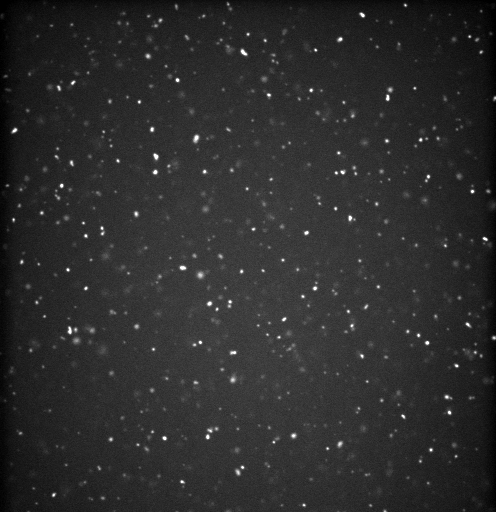

Supplement: Supplementary file 5 — Source data Fig. 3 [file 44318_2024_280_MOESM5_ESM.zip › SD figure 3/Fig3H/Condassay_TBK11_w2sd-mCherry_s3_t60.stk]

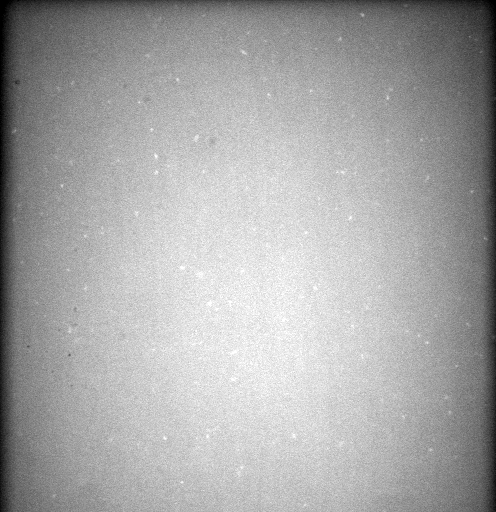

Supplement: Supplementary file 5 — Source data Fig. 3 [file 44318_2024_280_MOESM5_ESM.zip › SD figure 3/Fig3H/Condassay_TBK11_w1sd-GFP_s3_t60.stk]

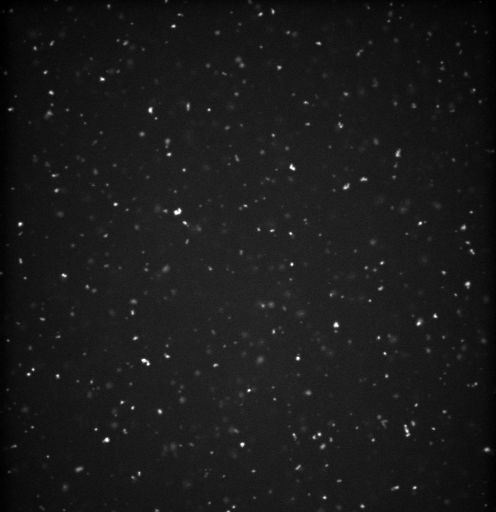

Supplement: Supplementary file 5 — Source data Fig. 3 [file 44318_2024_280_MOESM5_ESM.zip › SD figure 3/Fig3H/Condassay_TBK11_w2sd-mCherry_s4_t60.stk]

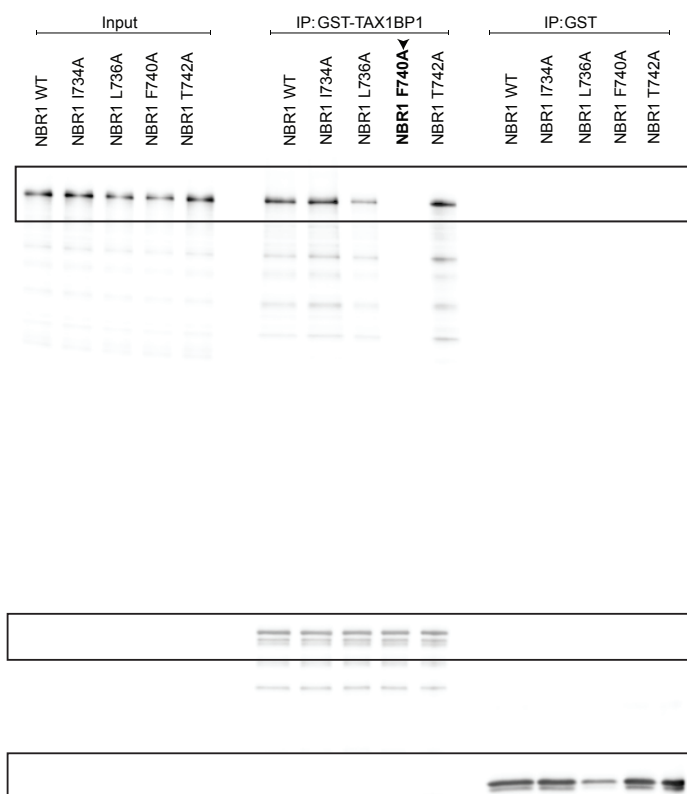

NBR1

TAX1BP1

anti GST

GST

Supplement: Supplementary file 6 — Source data Fig. 4 [file 44318_2024_280_MOESM6_ESM.zip › SD figure 4/Fig4H.pdf]

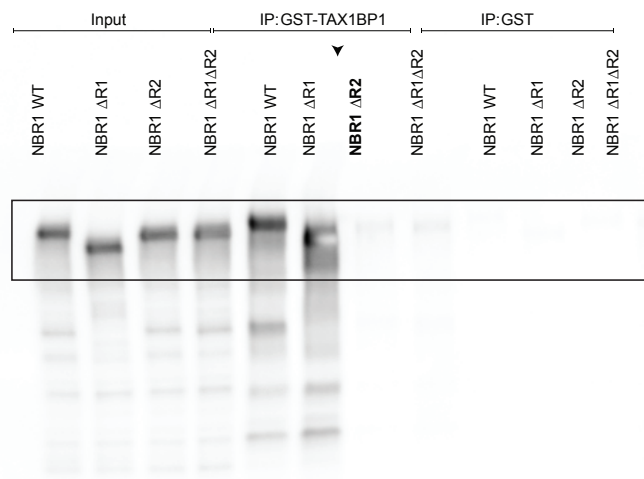

NBR1

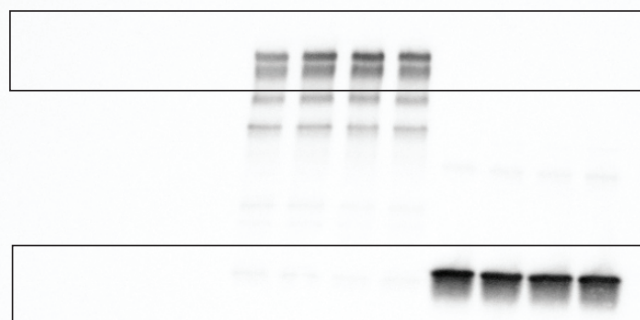

TAX1BP1

anti GST

GST

Supplement: Supplementary file 6 — Source data Fig. 4 [file 44318_2024_280_MOESM6_ESM.zip › SD figure 4/Fig4D.pdf]

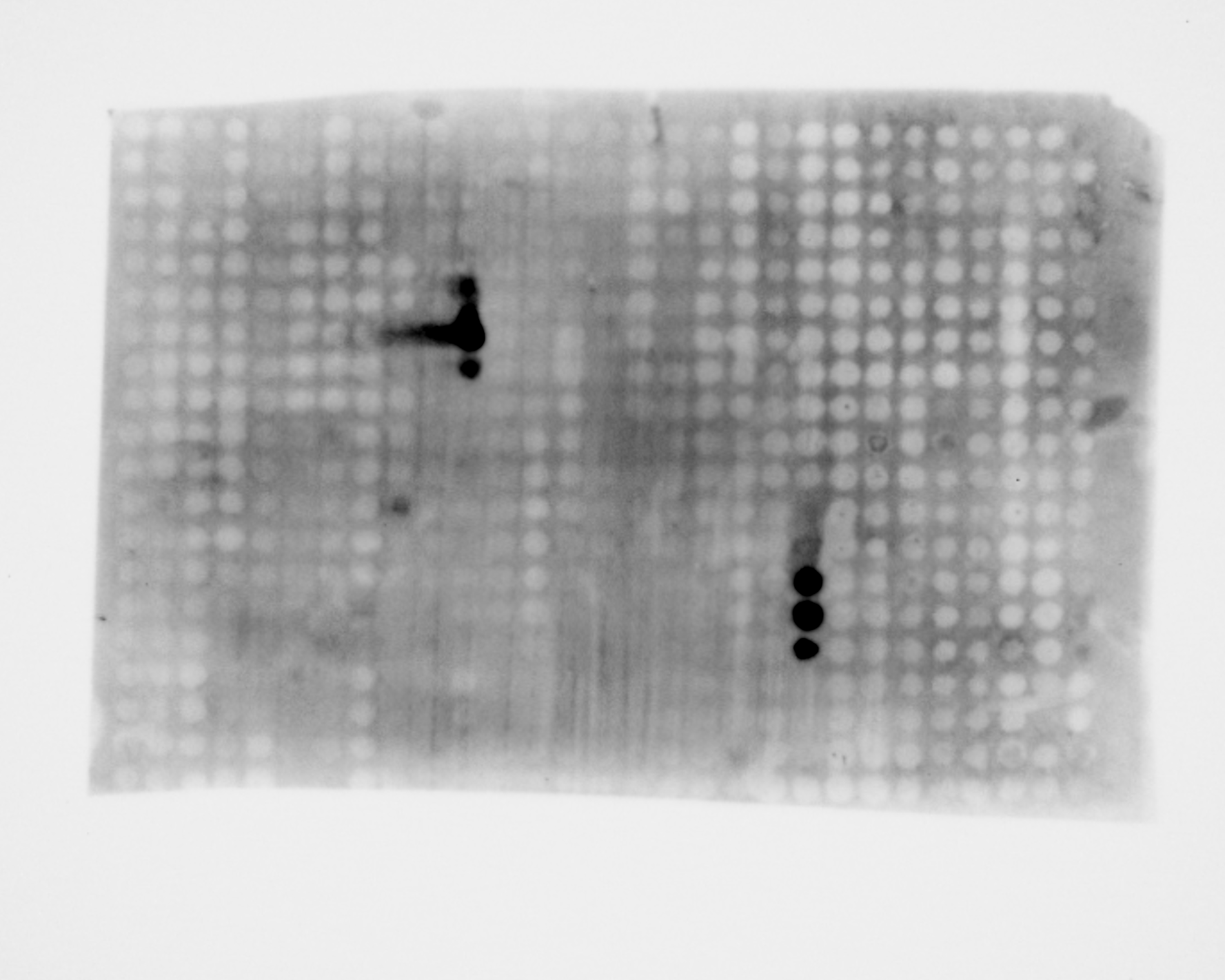

Supplement: Supplementary file 6 — Source data Fig. 4 [file 44318_2024_280_MOESM6_ESM.zip › SD figure 4/Fig4C.tif]

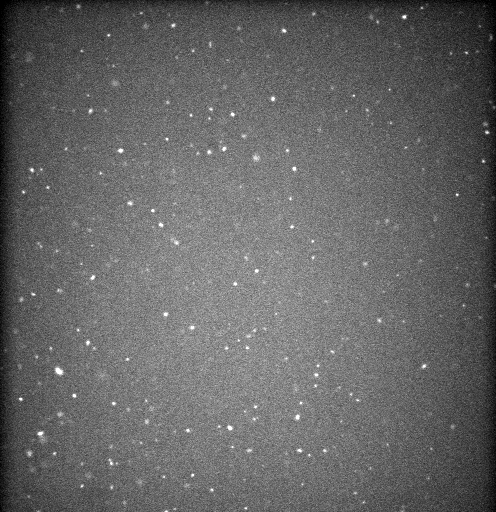

Supplement: Supplementary file 7 — Source data Fig. 5 [file 44318_2024_280_MOESM7_ESM.zip › SD figure 5/Fig5E/Condassay_2_w1sd-GFP_s3_t60.tif]

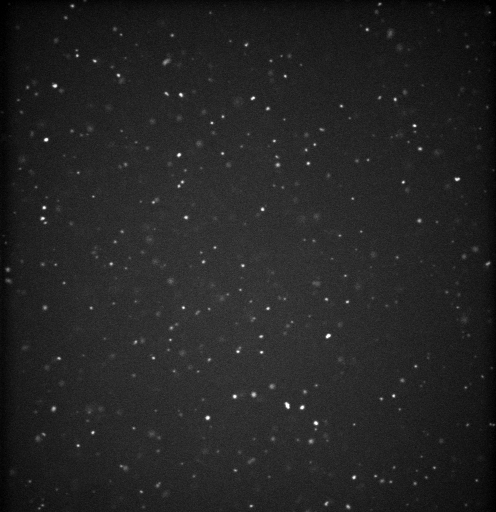

Supplement: Supplementary file 7 — Source data Fig. 5 [file 44318_2024_280_MOESM7_ESM.zip › SD figure 5/Fig5E/Condassay_2_w2sd-mCherry_s4_t60.tif]

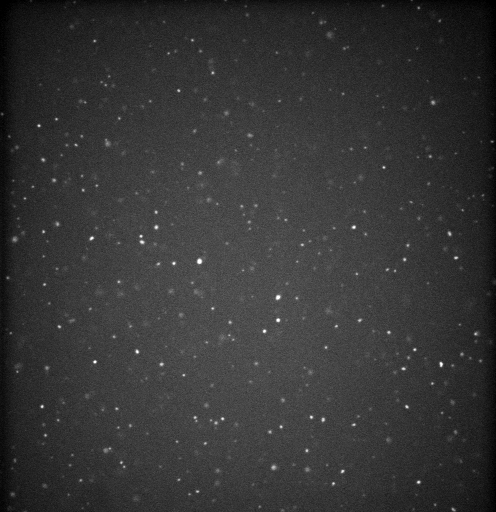

Supplement: Supplementary file 7 — Source data Fig. 5 [file 44318_2024_280_MOESM7_ESM.zip › SD figure 5/Fig5E/Condassay_2_w2sd-mCherry_s5_t60.tif]

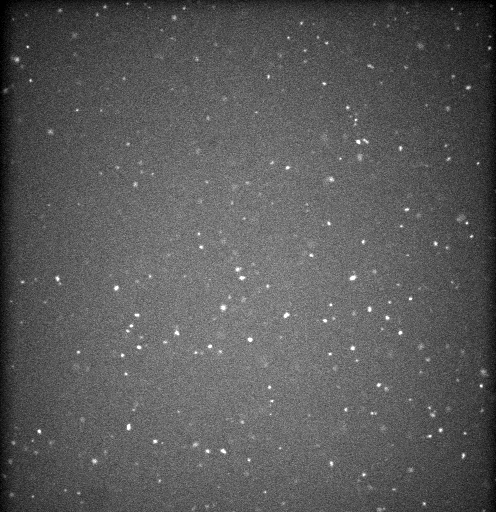

Supplement: Supplementary file 7 — Source data Fig. 5 [file 44318_2024_280_MOESM7_ESM.zip › SD figure 5/Fig5E/Condassay_2_w1sd-GFP_s1_t60.tif]

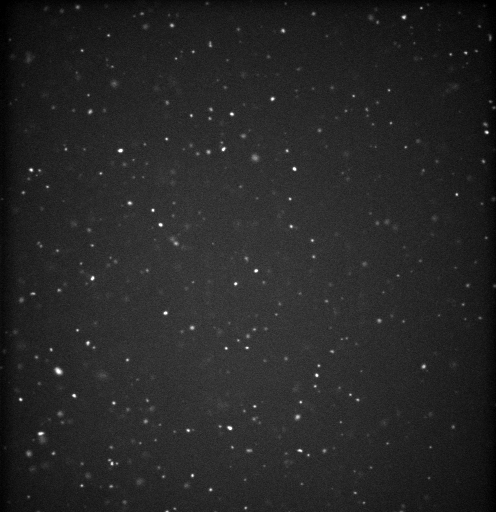

Supplement: Supplementary file 7 — Source data Fig. 5 [file 44318_2024_280_MOESM7_ESM.zip › SD figure 5/Fig5E/Condassay_2_w2sd-mCherry_s3_t60.tif]

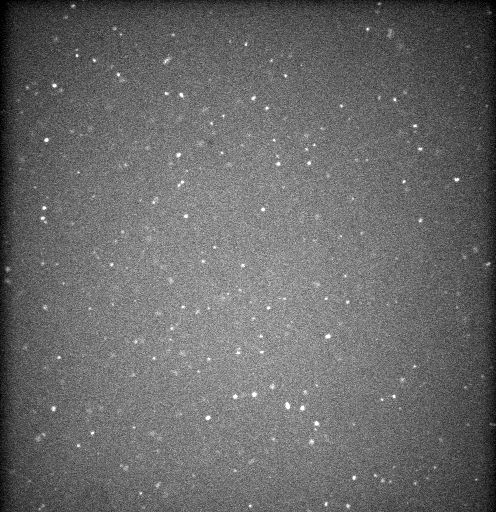

Supplement: Supplementary file 7 — Source data Fig. 5 [file 44318_2024_280_MOESM7_ESM.zip › SD figure 5/Fig5E/Condassay_2_w1sd-GFP_s4_t60.tif]

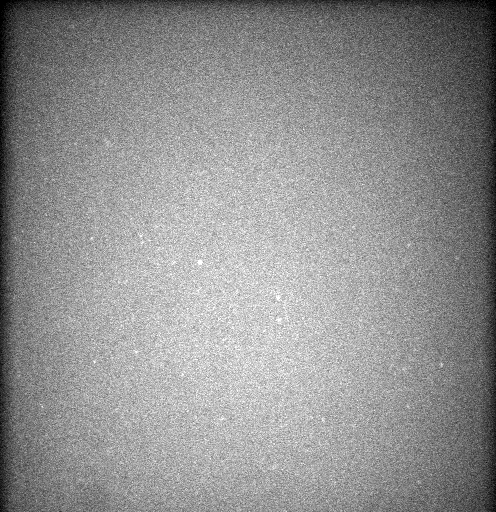

Supplement: Supplementary file 7 — Source data Fig. 5 [file 44318_2024_280_MOESM7_ESM.zip › SD figure 5/Fig5E/Condassay_2_w1sd-GFP_s5_t60.tif]

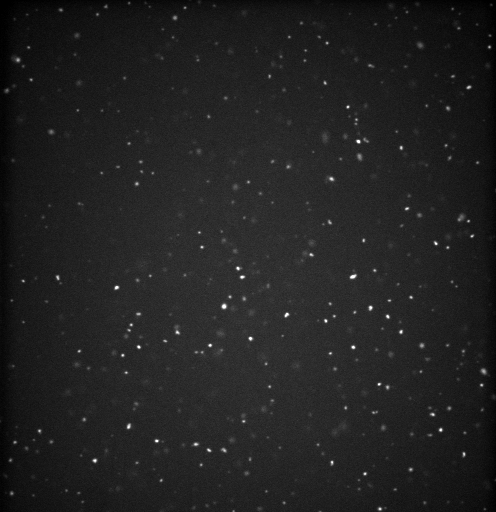

Supplement: Supplementary file 7 — Source data Fig. 5 [file 44318_2024_280_MOESM7_ESM.zip › SD figure 5/Fig5E/Condassay_2_w2sd-mCherry_s1_t60.tif]

| R-TAX1BP1 |   |   | R-TAX1BP1ΔN |   |   | R-TAX1BP1 L442A |   |   |           |
|-----------|---|---|-------------|---|---|-----------------|---|---|-----------|
| -         | + | + | -           | + | + | -               | + | + | Daycycle  |
| -         | - | - | -           | - | - | -               | - | - | VPS34 IN1 |

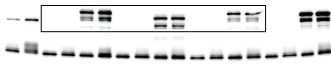

TAX1BP1

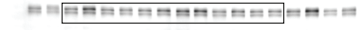

NBR1

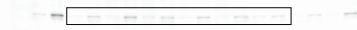

p62 p-S403

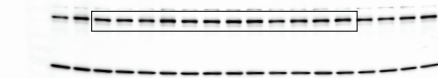

p62

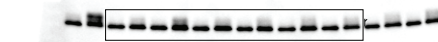

NAP1

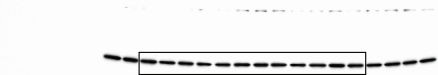

GAPDH

Supplement: Supplementary file 8 — Source data Fig. 6 [file 44318_2024_280_MOESM8_ESM.zip › SD figure 6/Fig6D.pdf]

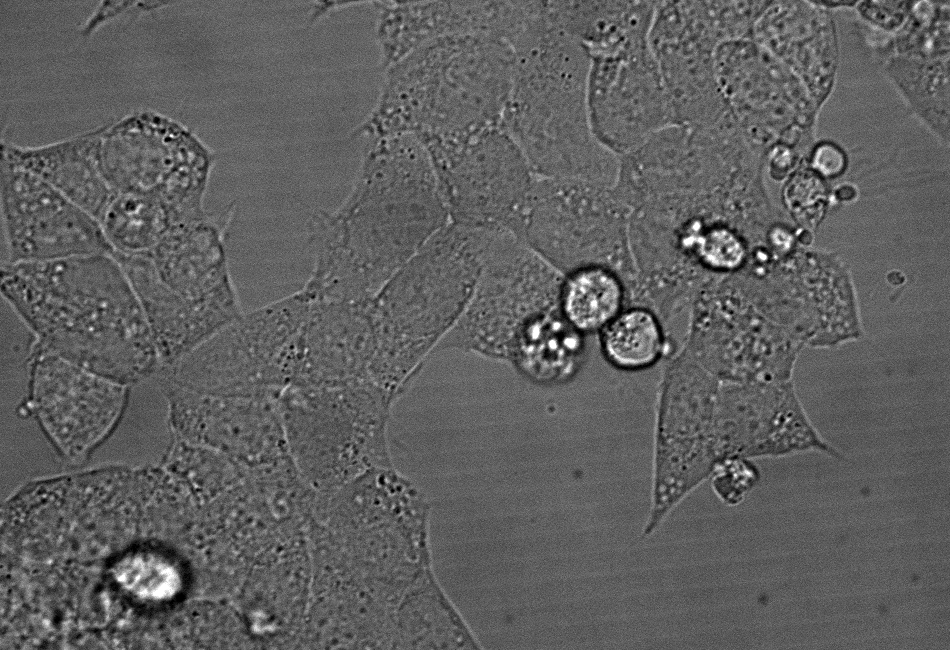

Supplement: Supplementary file 8 — Source data Fig. 6 [file 44318_2024_280_MOESM8_ESM.zip › SD figure 6/Fig6A/R-TAX1BP1_Dox_VPS.tif]

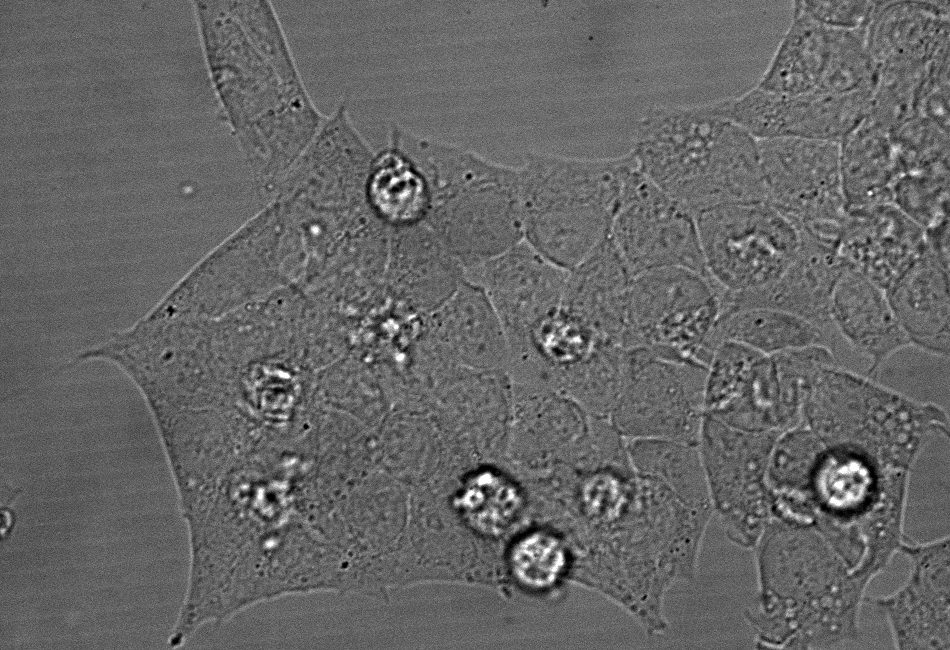

Supplement: Supplementary file 8 — Source data Fig. 6 [file 44318_2024_280_MOESM8_ESM.zip › SD figure 6/Fig6A/R-TAX1BP1_UT.tif]

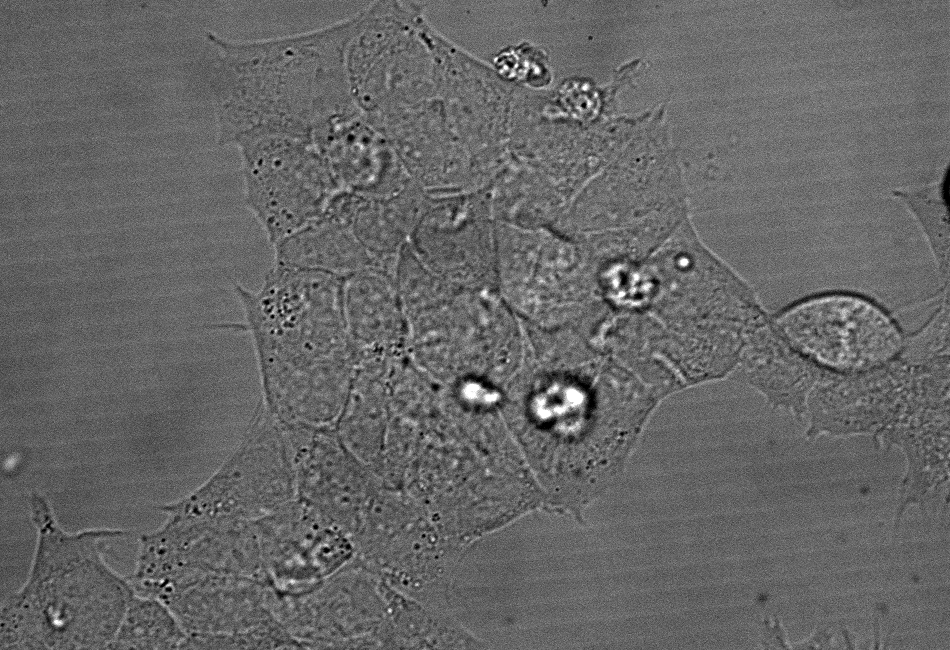

Supplement: Supplementary file 8 — Source data Fig. 6 [file 44318_2024_280_MOESM8_ESM.zip › SD figure 6/Fig6A/R-TAX1BP1-L442A_UT.tif]

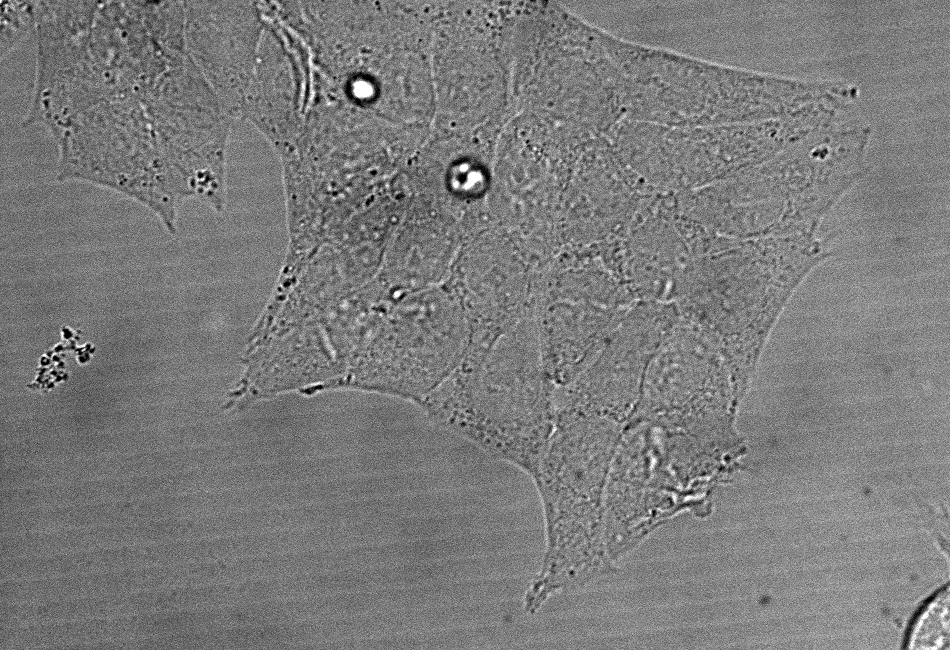

Supplement: Supplementary file 8 — Source data Fig. 6 [file 44318_2024_280_MOESM8_ESM.zip › SD figure 6/Fig6A/R-TAX1BP1_Dox.tif]

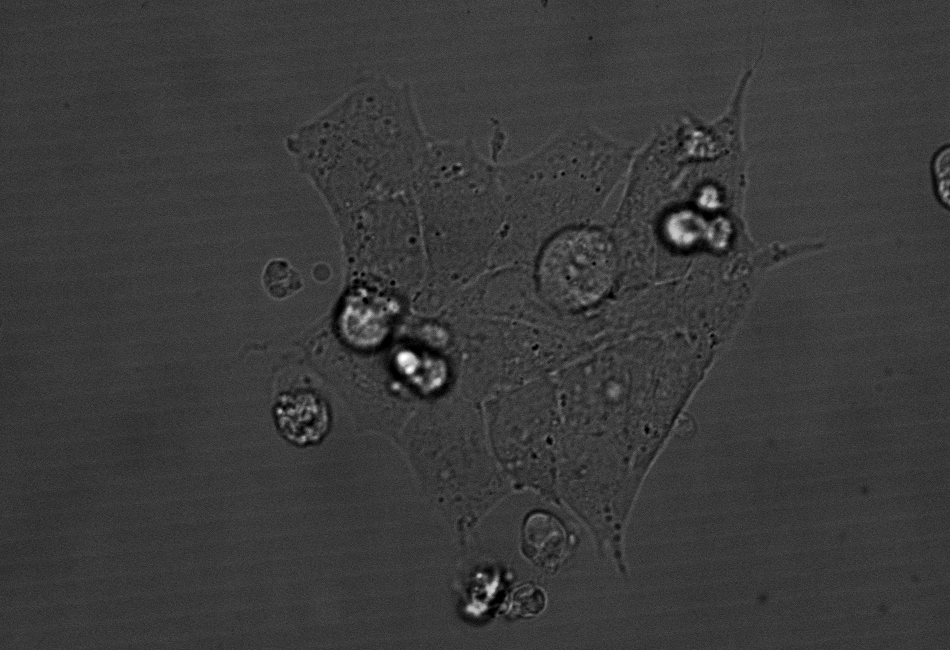

Supplement: Supplementary file 8 — Source data Fig. 6 [file 44318_2024_280_MOESM8_ESM.zip › SD figure 6/Fig6A/R-TAX1BP1ΓêåN_UT.tif]

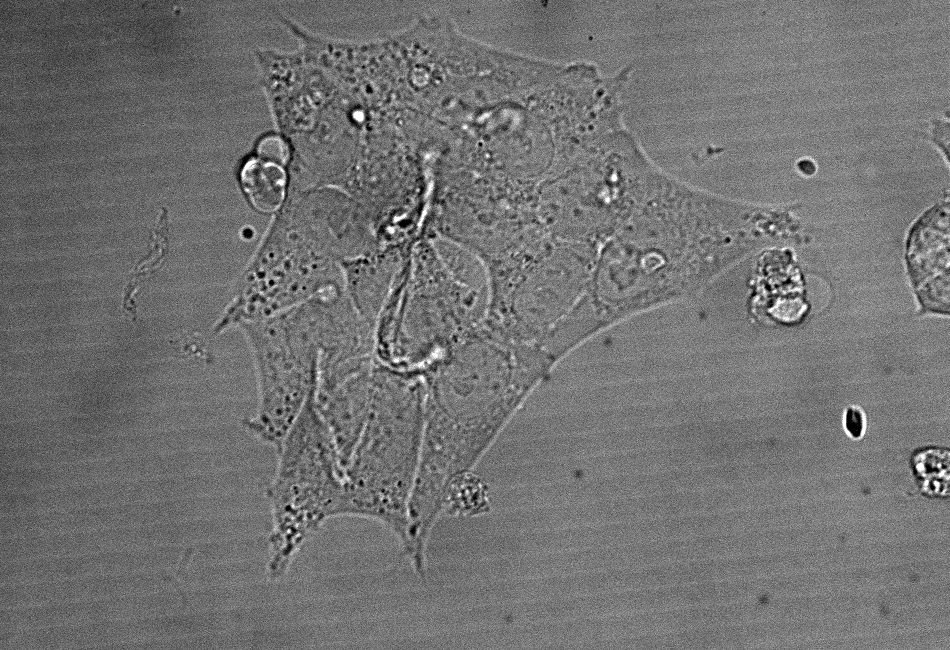

Supplement: Supplementary file 8 — Source data Fig. 6 [file 44318_2024_280_MOESM8_ESM.zip › SD figure 6/Fig6A/R-TAX1BP1-L442A_Dox_VPS.tif]

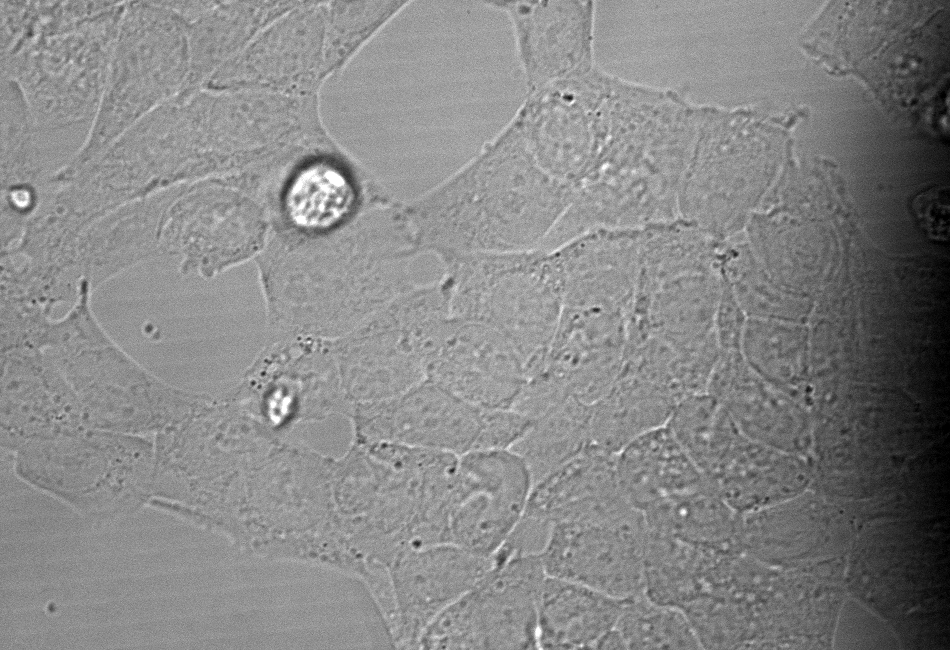

Supplement: Supplementary file 8 — Source data Fig. 6 [file 44318_2024_280_MOESM8_ESM.zip › SD figure 6/Fig6A/R-TAX1BP1ΓêåN_Dox.tif]

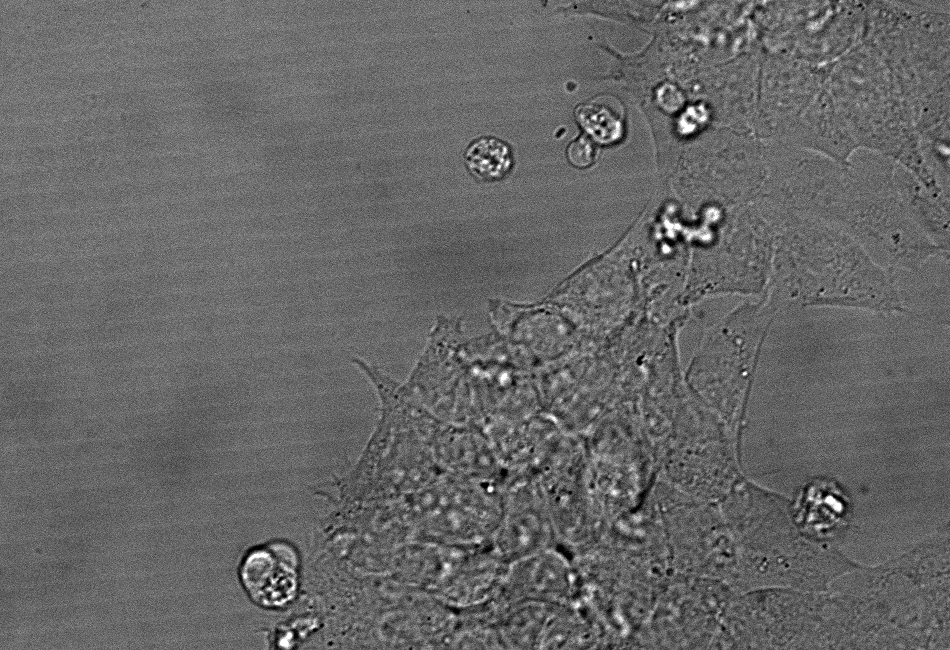

Supplement: Supplementary file 8 — Source data Fig. 6 [file 44318_2024_280_MOESM8_ESM.zip › SD figure 6/Fig6A/R-TAX1BP1-L442A_Dox.tif]

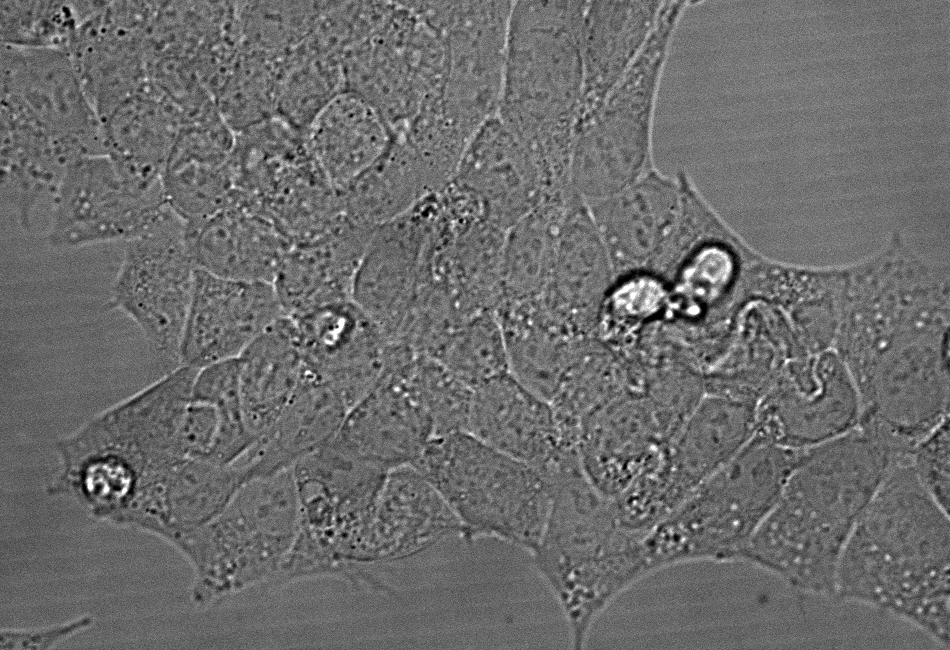

Supplement: Supplementary file 8 — Source data Fig. 6 [file 44318_2024_280_MOESM8_ESM.zip › SD figure 6/Fig6A/R-TAX1BP1ΓêåN_Dox_VPS.tif]

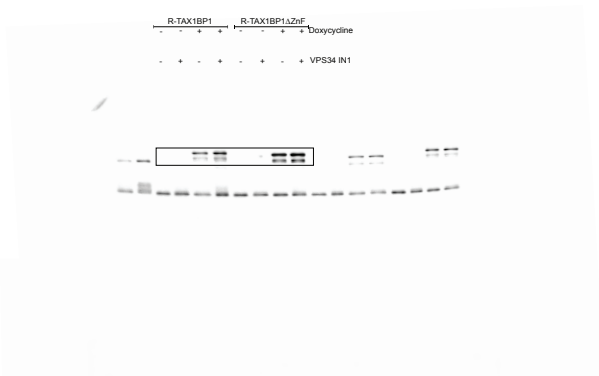

TAX1BP1

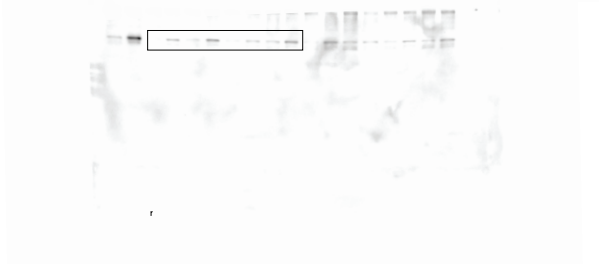

p62 p-S403

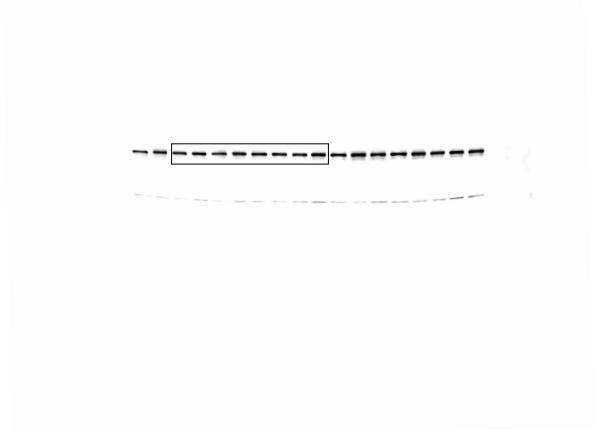

p62

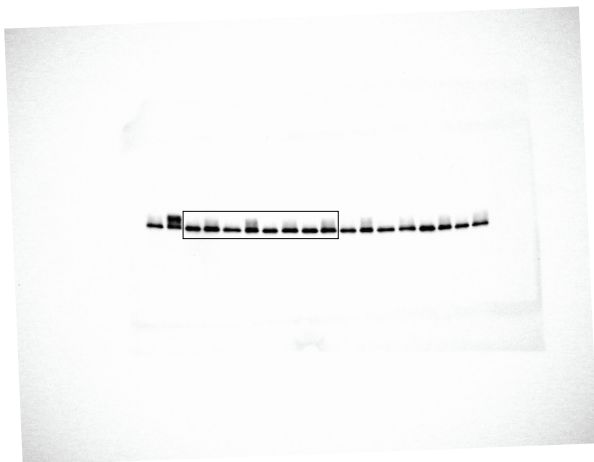

NAP1

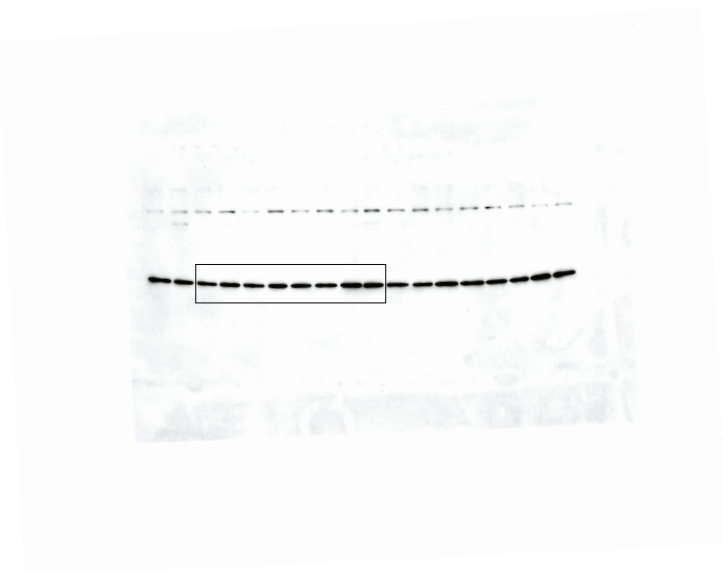

GAPDH

Supplement: Supplementary file 9 — Source data Fig. 7 [file 44318_2024_280_MOESM9_ESM.zip › SD figure 7/Fig7F.pdf]

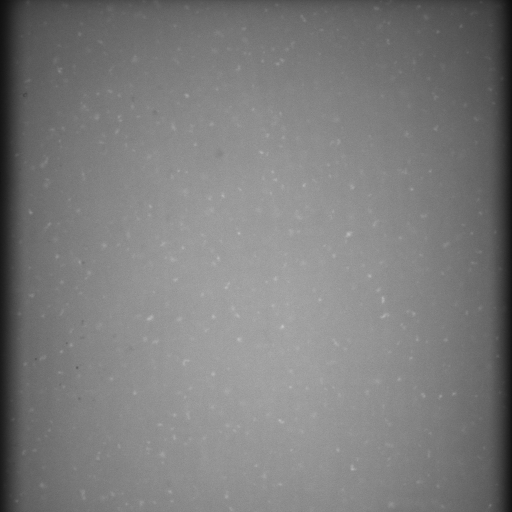

Supplement: Supplementary file 9 — Source data Fig. 7 [file 44318_2024_280_MOESM9_ESM.zip › SD figure 7/Fig7B/Condassay1_w1sd-GFP_s4_t15.stk]

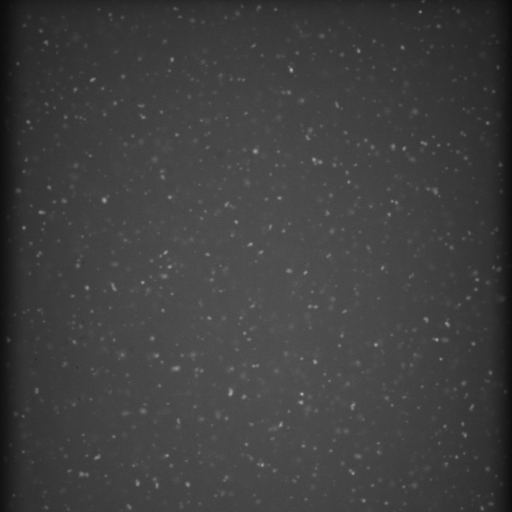

Supplement: Supplementary file 9 — Source data Fig. 7 [file 44318_2024_280_MOESM9_ESM.zip › SD figure 7/Fig7B/Condassay1_w1sd-GFP_s5_t15.stk]

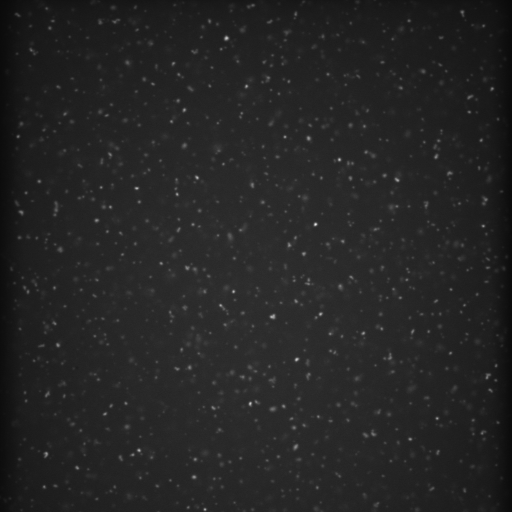

Supplement: Supplementary file 9 — Source data Fig. 7 [file 44318_2024_280_MOESM9_ESM.zip › SD figure 7/Fig7B/Condassay1_w2sd-mCherry_s2_t15.stk]

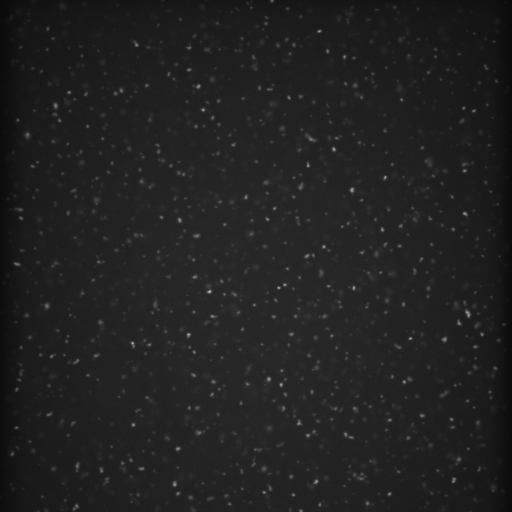

Supplement: Supplementary file 9 — Source data Fig. 7 [file 44318_2024_280_MOESM9_ESM.zip › SD figure 7/Fig7B/Condassay1_w2sd-mCherry_s3_t15.stk]

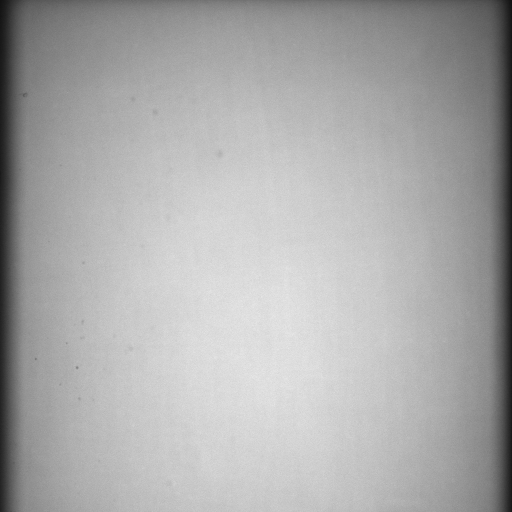

Supplement: Supplementary file 9 — Source data Fig. 7 [file 44318_2024_280_MOESM9_ESM.zip › SD figure 7/Fig7B/Condassay1_w1sd-GFP_s3_t15.stk]

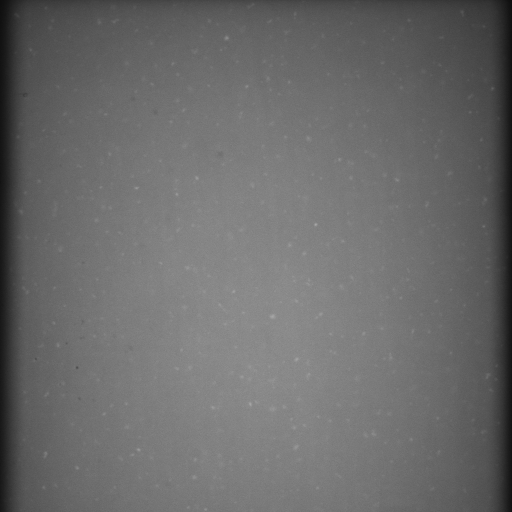

Supplement: Supplementary file 9 — Source data Fig. 7 [file 44318_2024_280_MOESM9_ESM.zip › SD figure 7/Fig7B/Condassay1_w1sd-GFP_s2_t15.stk]

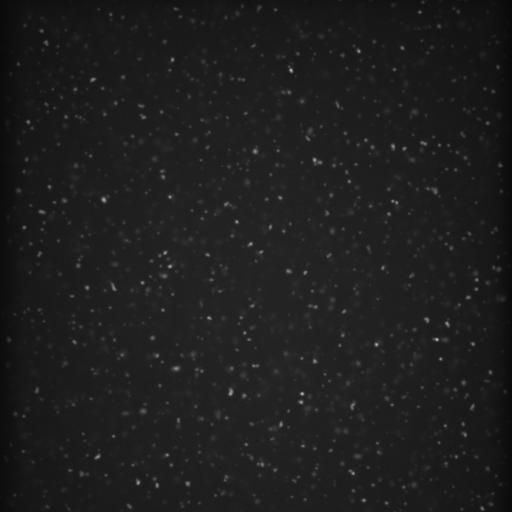

Supplement: Supplementary file 9 — Source data Fig. 7 [file 44318_2024_280_MOESM9_ESM.zip › SD figure 7/Fig7B/Condassay1_w2sd-mCherry_s5_t15.stk]

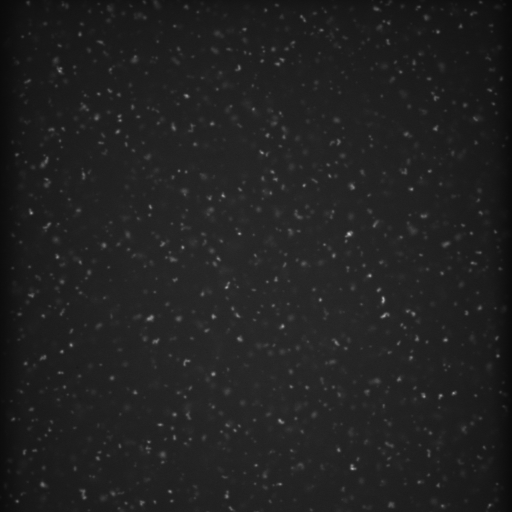

Supplement: Supplementary file 9 — Source data Fig. 7 [file 44318_2024_280_MOESM9_ESM.zip › SD figure 7/Fig7B/Condassay1_w2sd-mCherry_s4_t15.stk]

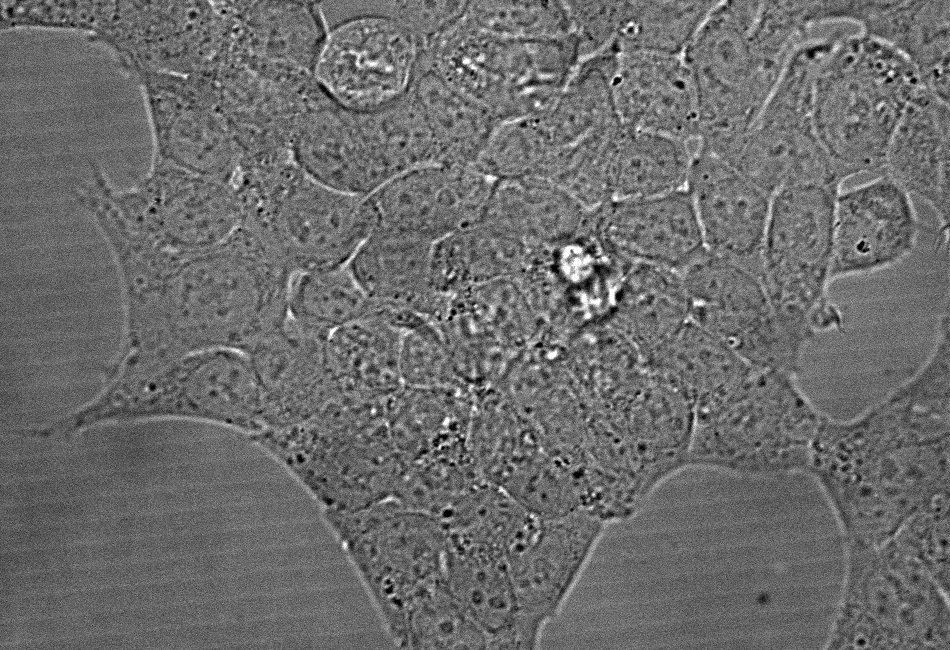

Supplement: Supplementary file 9 — Source data Fig. 7 [file 44318_2024_280_MOESM9_ESM.zip › SD figure 7/Fig7D/R-TAX1BP1ΓêåZnF_Dox.tif]

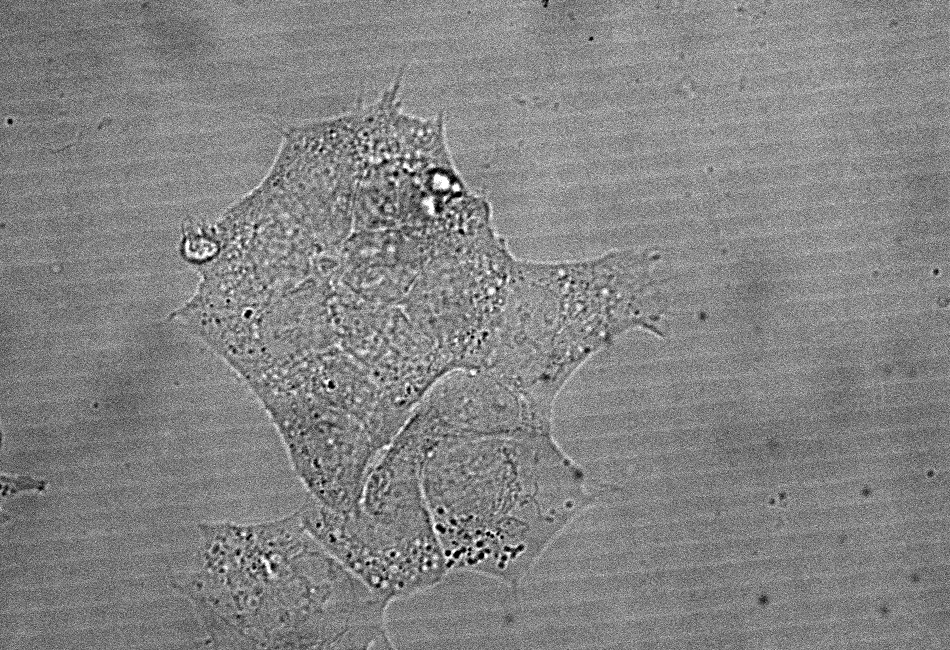

Supplement: Supplementary file 9 — Source data Fig. 7 [file 44318_2024_280_MOESM9_ESM.zip › SD figure 7/Fig7D/R-TAX1BP1ΓêåZnF_UT.tif]

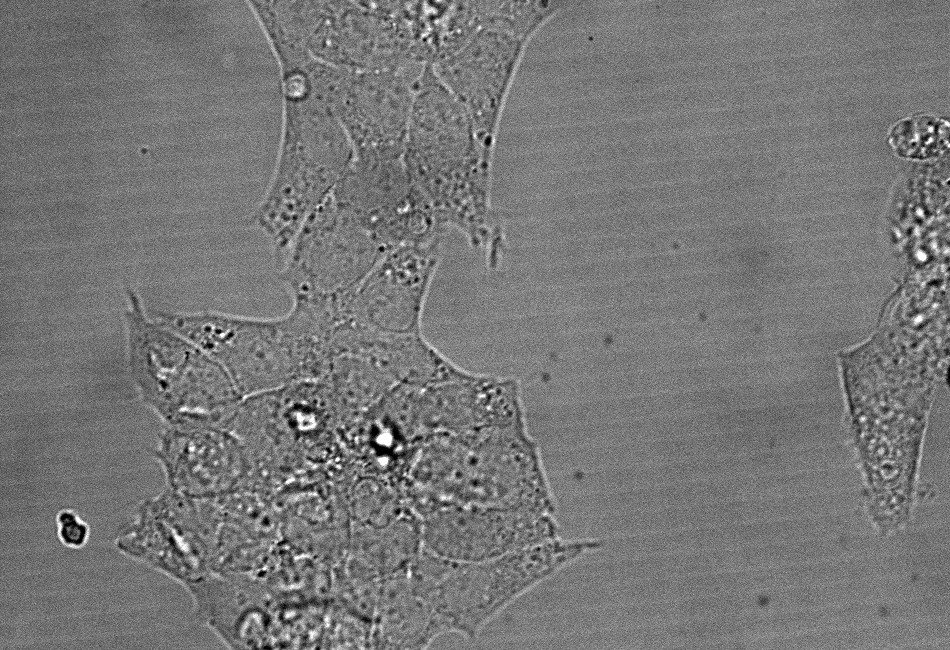

Supplement: Supplementary file 9 — Source data Fig. 7 [file 44318_2024_280_MOESM9_ESM.zip › SD figure 7/Fig7D/R-TAX1BP1ΓêåZnF_Dox_VPS.tif]

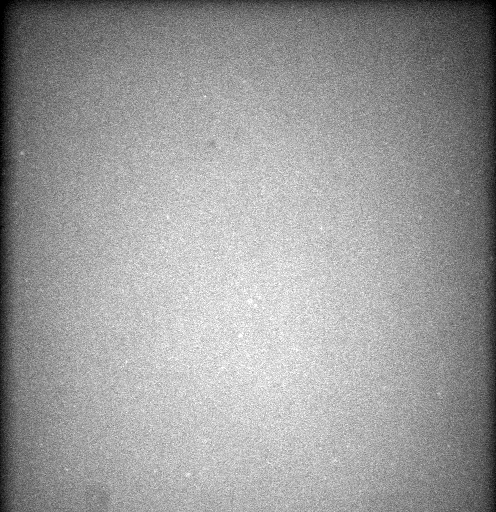

Supplement: Supplementary file 9 — Source data Fig. 7 [file 44318_2024_280_MOESM9_ESM.zip › SD figure 7/Fig7J/GST-4xUB_withGABA_30min_GFP.tif]

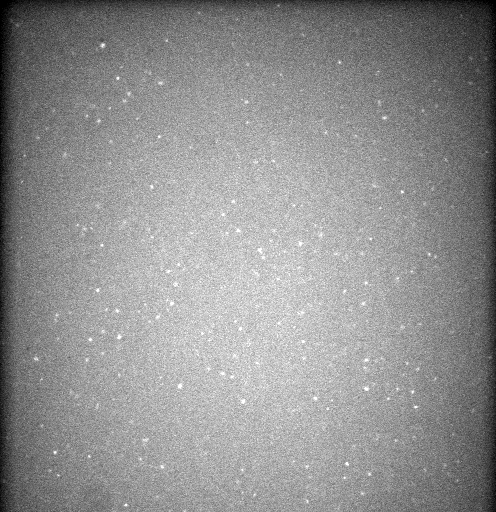

Supplement: Supplementary file 9 — Source data Fig. 7 [file 44318_2024_280_MOESM9_ESM.zip › SD figure 7/Fig7J/GST-4xUB_noGABA_30min_GFP.tif]

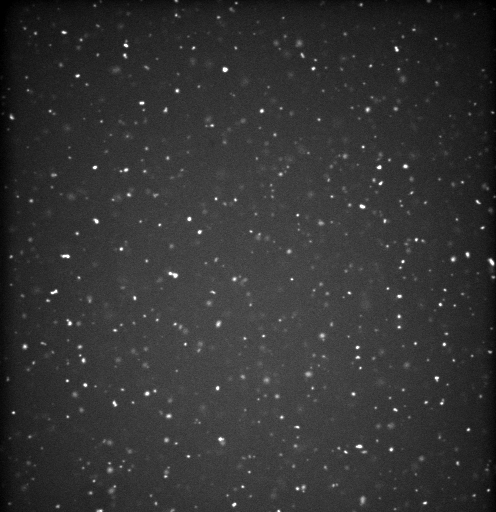

Supplement: Supplementary file 9 — Source data Fig. 7 [file 44318_2024_280_MOESM9_ESM.zip › SD figure 7/Fig7J/GST-8xUB_withGABA_30min_mCh.tif]

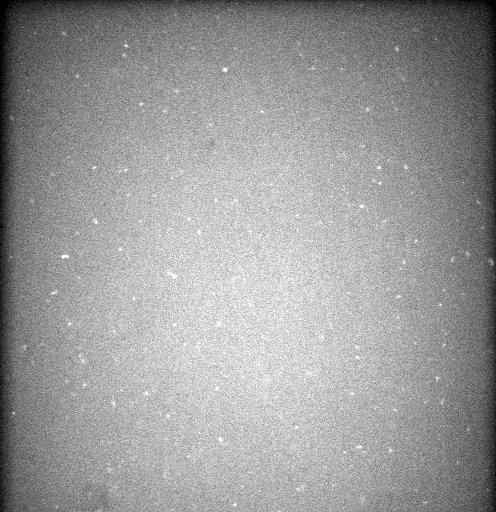

Supplement: Supplementary file 9 — Source data Fig. 7 [file 44318_2024_280_MOESM9_ESM.zip › SD figure 7/Fig7J/GST-8xUB_withGABA_30min_GFP.tif]

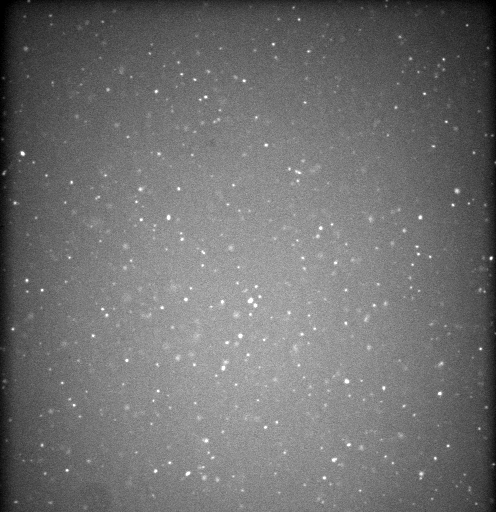

Supplement: Supplementary file 9 — Source data Fig. 7 [file 44318_2024_280_MOESM9_ESM.zip › SD figure 7/Fig7J/GST-4xUB_withGABA_30min_mCh.tif]

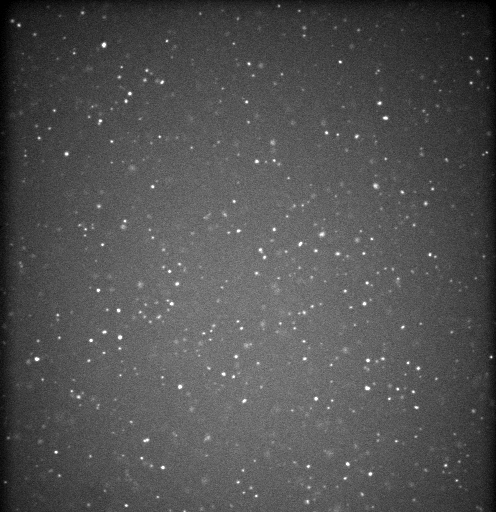

Supplement: Supplementary file 9 — Source data Fig. 7 [file 44318_2024_280_MOESM9_ESM.zip › SD figure 7/Fig7J/GST-4xUB_noGABA_30min_mCh.tif]

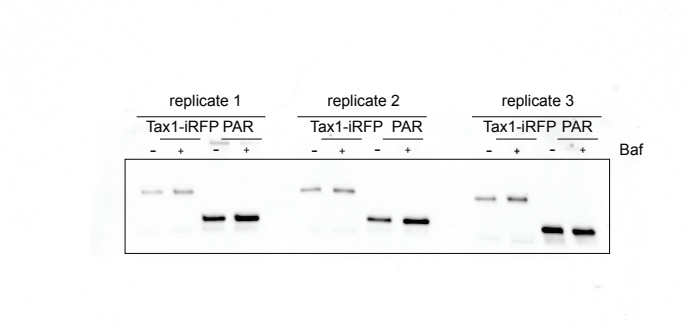

TAX1BP1

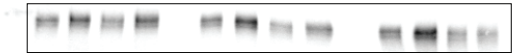

NBR1

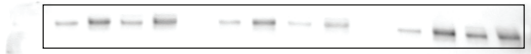

p62 p-S403

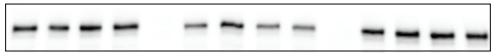

p62

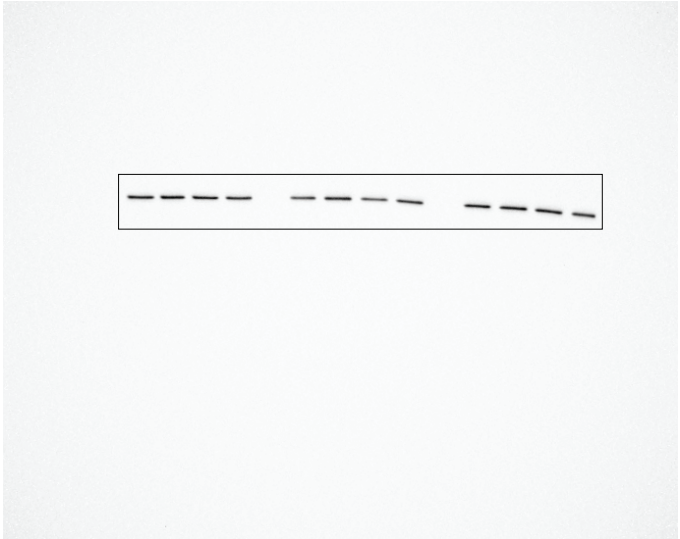

GAPDH

Supplement: Supplementary file 10 — EV and Appendix Figure Source Data [file 44318_2024_280_MOESM10_ESM.zip › SD FigEV_Appendix/FigEV1A.pdf]

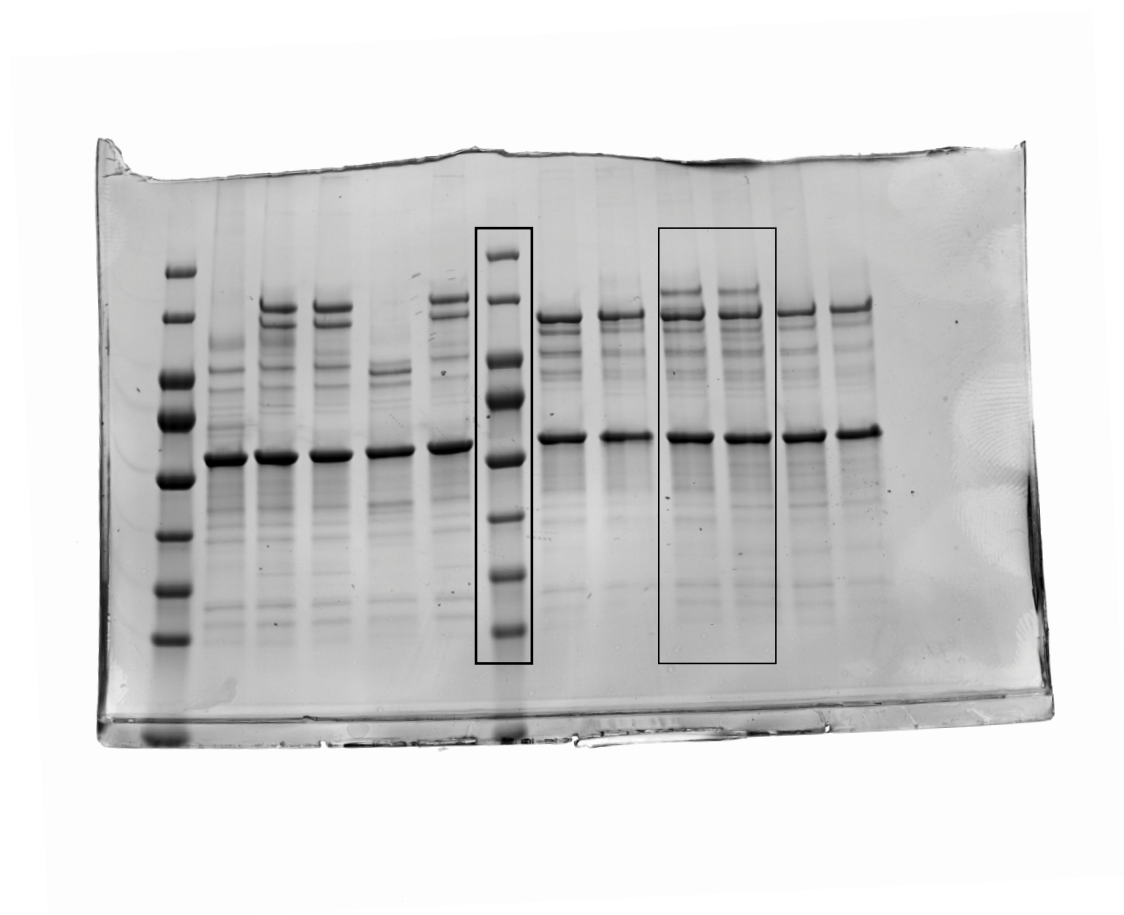

Supplement: Supplementary file 10 — EV and Appendix Figure Source Data [file 44318_2024_280_MOESM10_ESM.zip › SD FigEV_Appendix/FigEV2H.pdf]

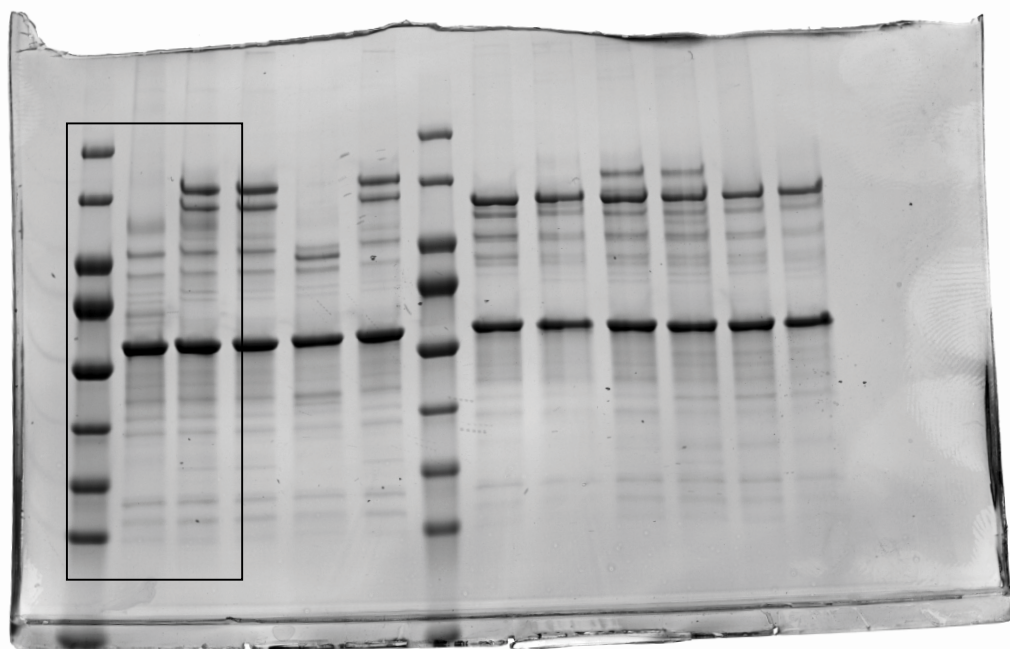

Supplement: Supplementary file 10 — EV and Appendix Figure Source Data [file 44318_2024_280_MOESM10_ESM.zip › SD FigEV_Appendix/FigEV2G.pdf]

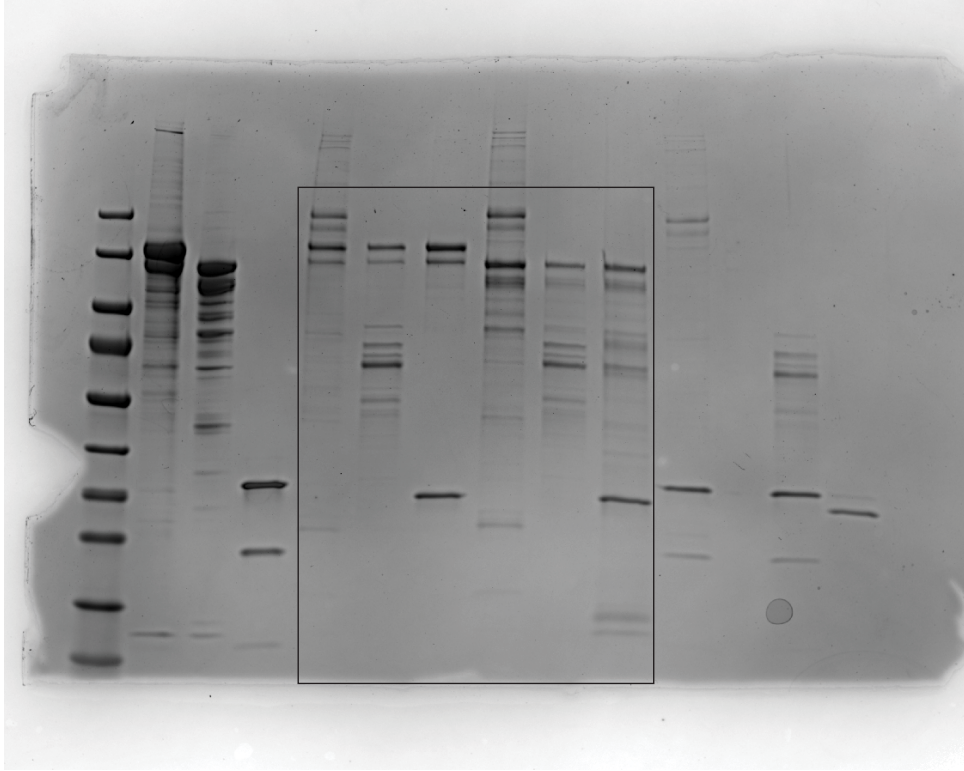

Supplement: Supplementary file 10 — EV and Appendix Figure Source Data [file 44318_2024_280_MOESM10_ESM.zip › SD FigEV_Appendix/FigEV3C.pdf]

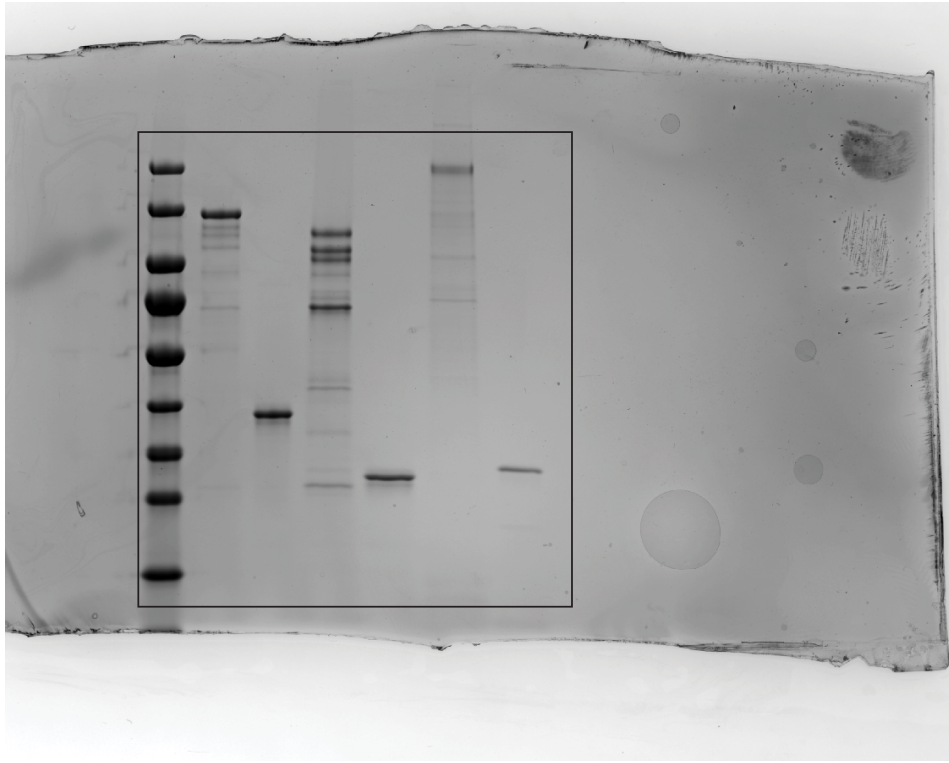

Supplement: Supplementary file 10 — EV and Appendix Figure Source Data [file 44318_2024_280_MOESM10_ESM.zip › SD FigEV_Appendix/FigEV3A.pdf]

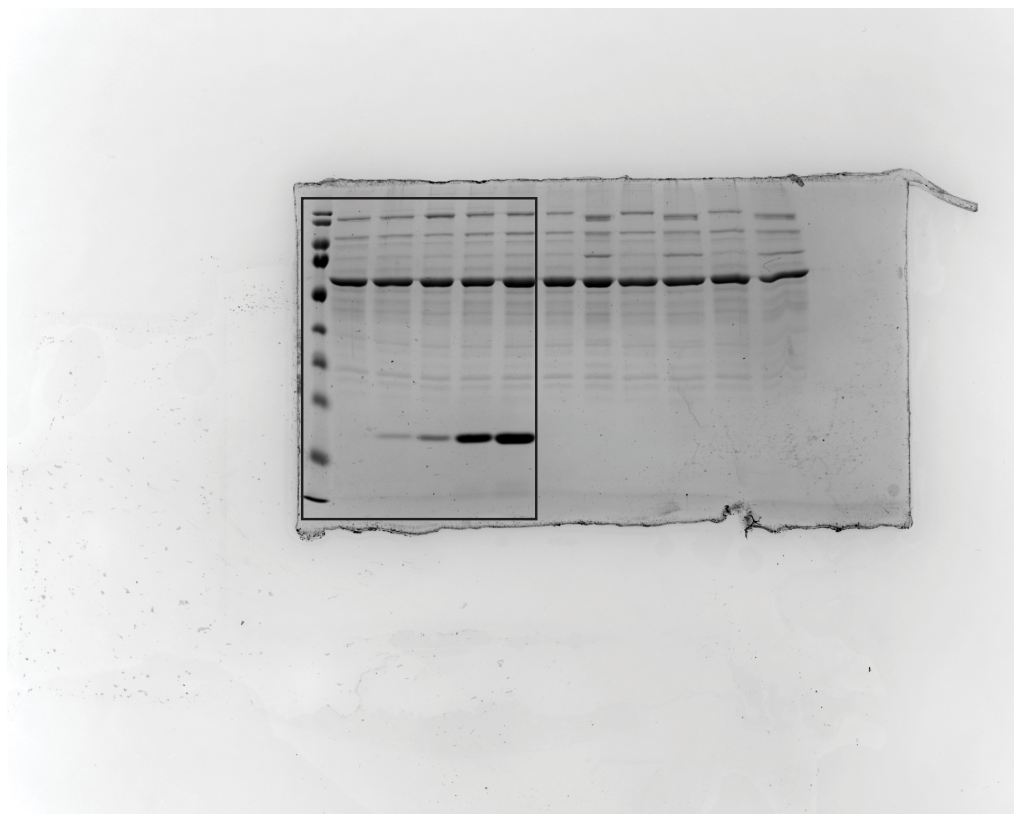

Supplement: Supplementary file 10 — EV and Appendix Figure Source Data [file 44318_2024_280_MOESM10_ESM.zip › SD FigEV_Appendix/Appendix_FigS2B.pdf]

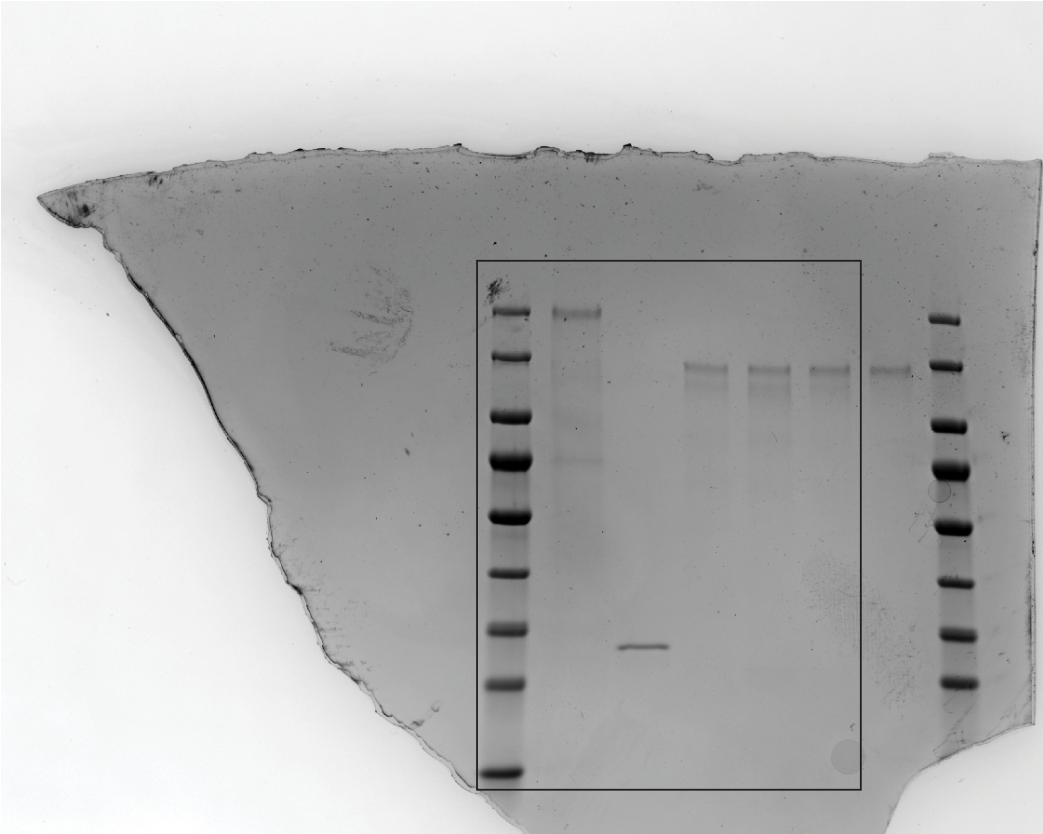

Supplement: Supplementary file 10 — EV and Appendix Figure Source Data [file 44318_2024_280_MOESM10_ESM.zip › SD FigEV_Appendix/FigEV3E.pdf]

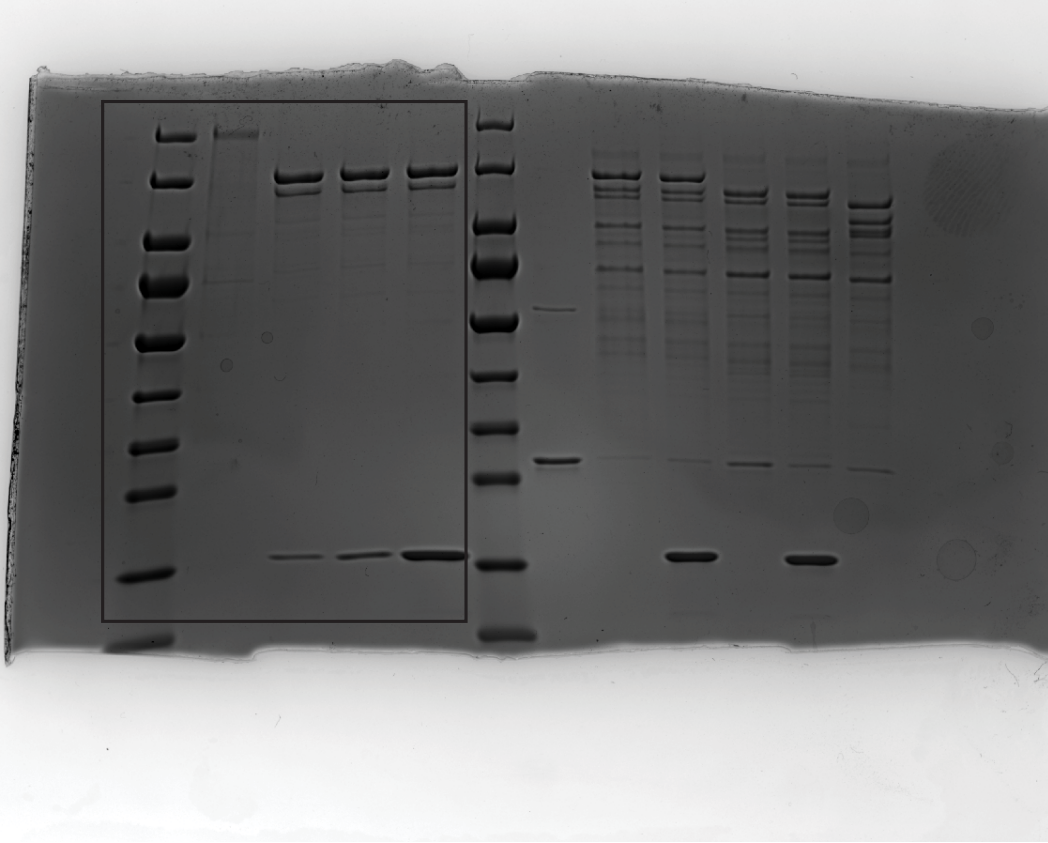

Supplement: Supplementary file 10 — EV and Appendix Figure Source Data [file 44318_2024_280_MOESM10_ESM.zip › SD FigEV_Appendix/Appendix_FigS2A.pdf]

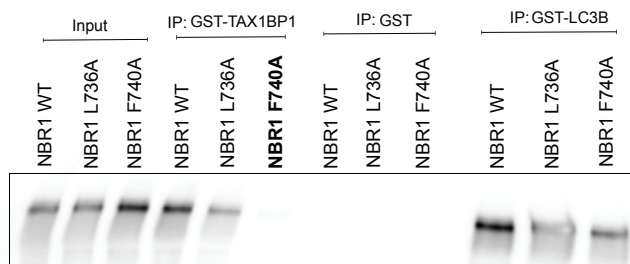

NBR1

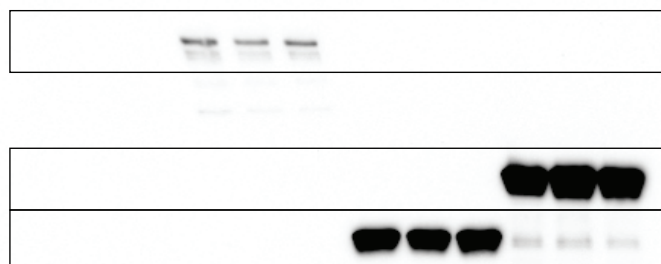

TAX1BP1

LC3B      anti GST

GST

Supplement: Supplementary file 10 — EV and Appendix Figure Source Data [file 44318_2024_280_MOESM10_ESM.zip › SD FigEV_Appendix/FigEV3F.pdf]

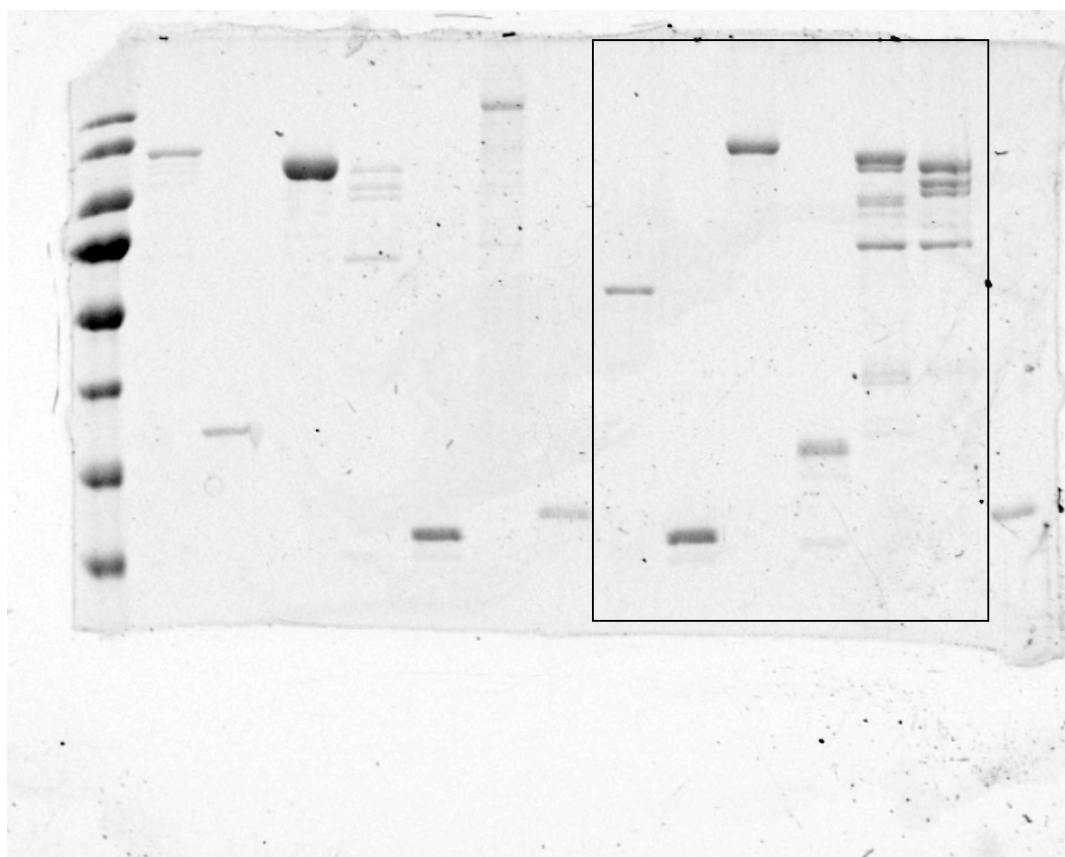

Supplement: Supplementary file 10 — EV and Appendix Figure Source Data [file 44318_2024_280_MOESM10_ESM.zip › SD FigEV_Appendix/FigEV5C.pdf]

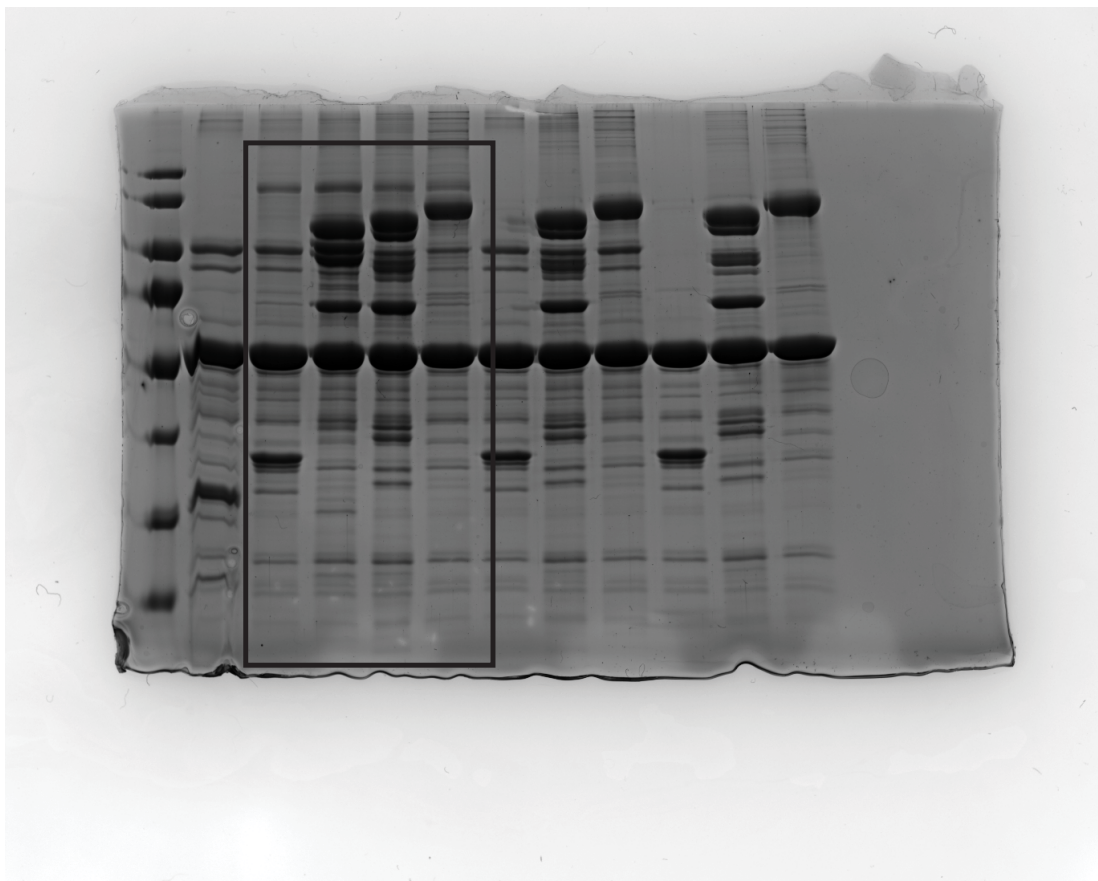

Supplement: Supplementary file 10 — EV and Appendix Figure Source Data [file 44318_2024_280_MOESM10_ESM.zip › SD FigEV_Appendix/FigEV5D.pdf]

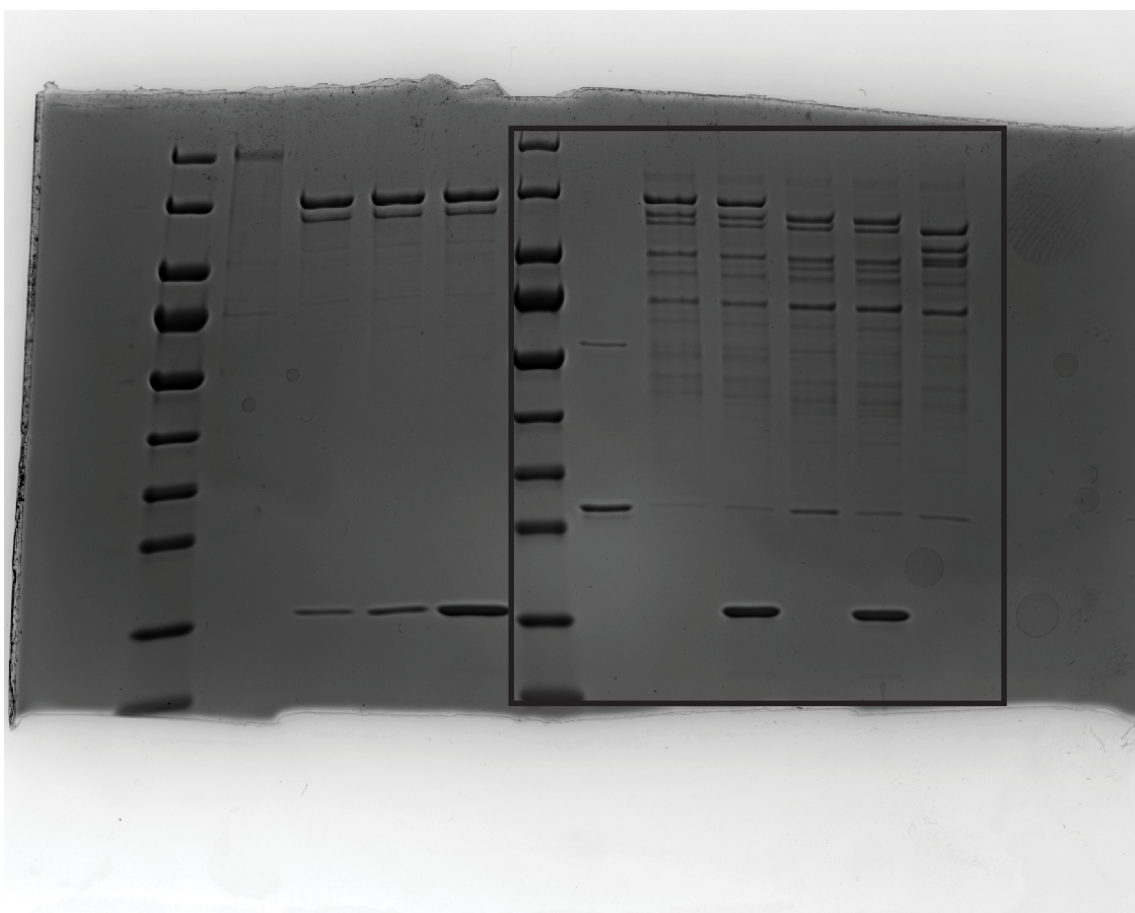

Supplement: Supplementary file 10 — EV and Appendix Figure Source Data [file 44318_2024_280_MOESM10_ESM.zip › SD FigEV_Appendix/FigEV5E.pdf]

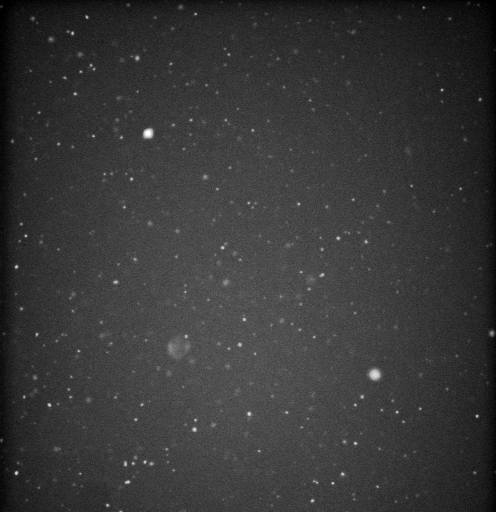

Supplement: Supplementary file 10 — EV and Appendix Figure Source Data [file 44318_2024_280_MOESM10_ESM.zip › SD FigEV_Appendix/FigEV2E/-NAP1_mCh.tif]

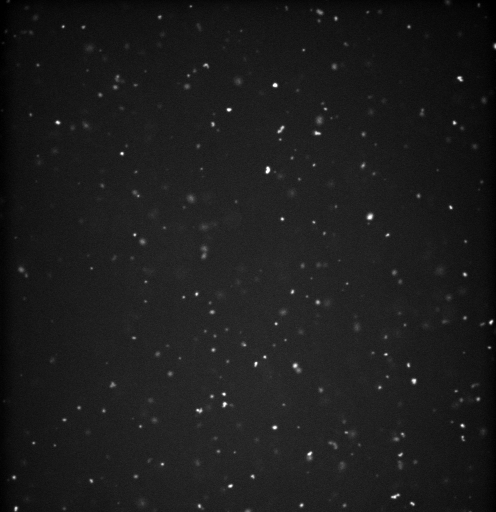

Supplement: Supplementary file 10 — EV and Appendix Figure Source Data [file 44318_2024_280_MOESM10_ESM.zip › SD FigEV_Appendix/FigEV2E/+NAP1_mCh.tif]

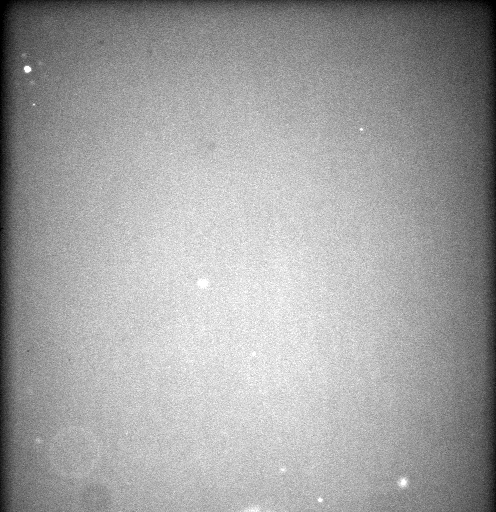

Supplement: Supplementary file 10 — EV and Appendix Figure Source Data [file 44318_2024_280_MOESM10_ESM.zip › SD FigEV_Appendix/FigEV2E/-NAP1_GFP.tif]

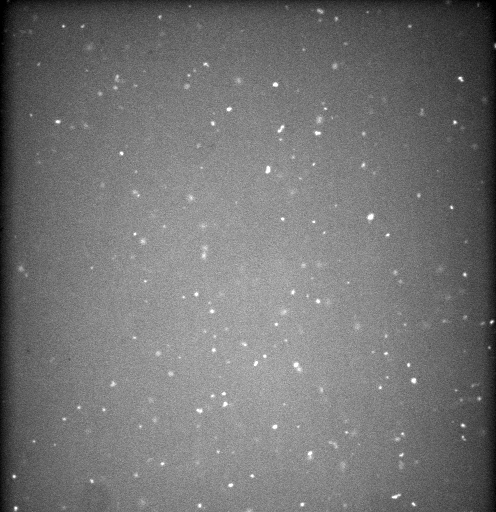

Supplement: Supplementary file 10 — EV and Appendix Figure Source Data [file 44318_2024_280_MOESM10_ESM.zip › SD FigEV_Appendix/FigEV2E/+NAP1_GFP.tif]
