# Supplementary material for: Recruitment of autophagy initiator TAX1BP1 advances aggrephagy from cargo collection to sequestration
Source: EMBO J. 2024 Oct 24;43(23):9. doi: 10.1038/s44318-024-00280-5 (PMC11611905; doi:10.1038/s44318-024-00280-5)
Supplement: Supplementary file 11 — Expanded View Figures [file 44318_2024_280_MOESM11_ESM.pdf]

Expanded View Figures

Figure EV1. TAX1BP1 is not a constitutive component of p62 condensates in cells.

(**A**) Western blot for the validation of the CRISPR knock-in cell line (PAR = parental cell line). (**B**) Quantification of (**A**). The increase between untreated and Bafilomycin treatment was plotted and compared to the parental cell line (PAR). (**C**) Representative immunofluorescence images of HAP1 WT +/- VPS34 IN1, ATG14 KO, ATG7 KO and FIP200 KO cells stained for p62 and TAX1BP1 (scale bar = 20 µm). Line scan of fluorescence intensities of p62 and TAX1BP1 to the right side of the corresponding images. The data in (**B**) shows the mean +/− s.d. from three independent experiments. Two-way ANOVA with Sidak's multiple comparison test was performed in (**B**). Source data are available online for this figure.

 © The Author(s)

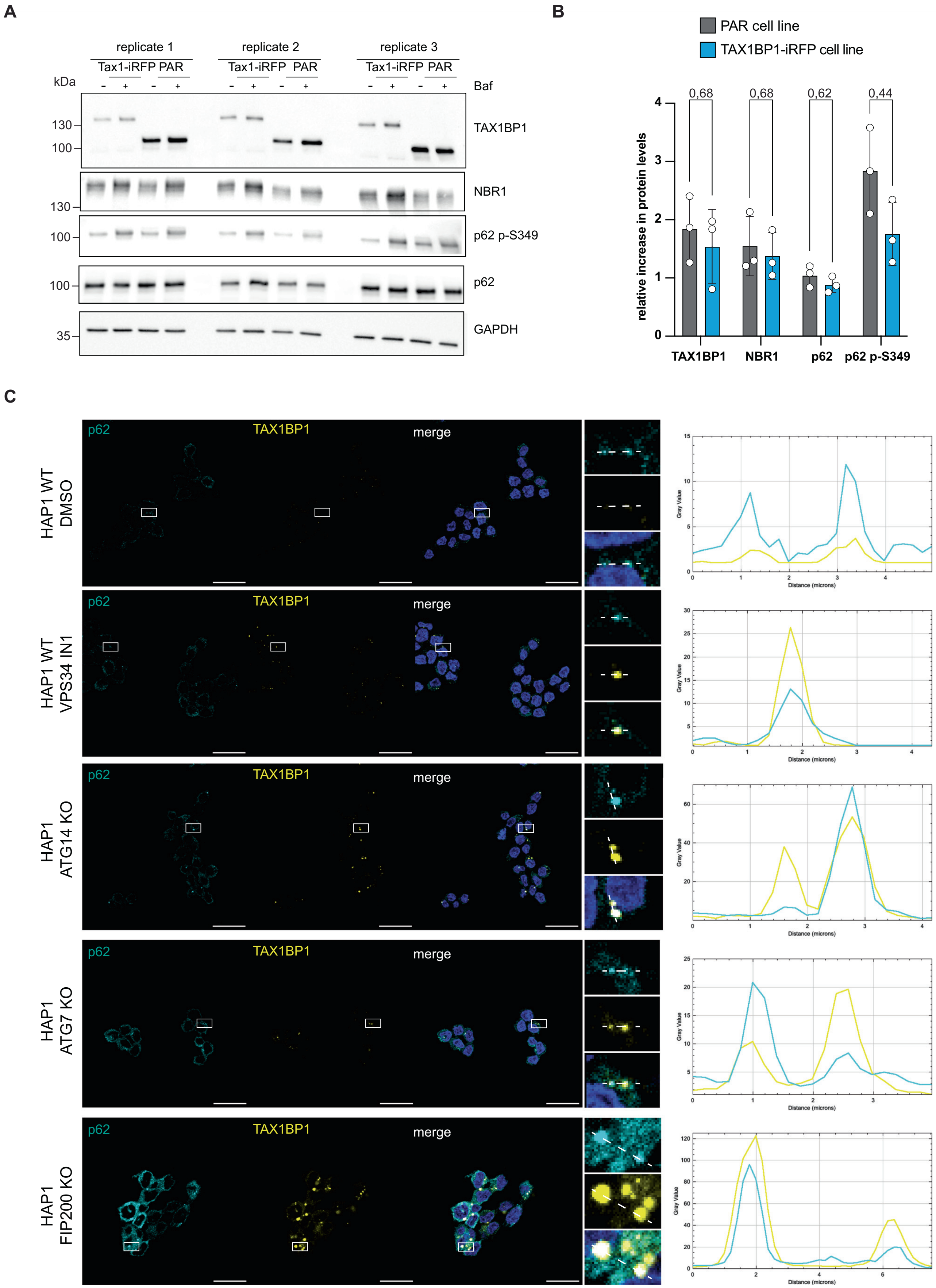
A
replicate 1
replicate 2
replicate 3
Tax1-iRFP PAR
kDa
Baf
TAX1BP1
NBR1
p62 p-S349
p62
GAPDH
130
100
35
B
PAR cell line
TAX1BP1-iRFP cell line
0,68
0,68
0,62
0,44
relative increase in protein levels
TAX1BP1
NBR1
p62
p62 p-S349
C
p62
TAX1BP1
merge
HAP1 WT DMSO
HAP1 WT VPS34 IN1
HAP1 ATG14 KO
HAP1 ATG7 KO
HAP1 FIP200 KO
Gray Value
Distance (microns)

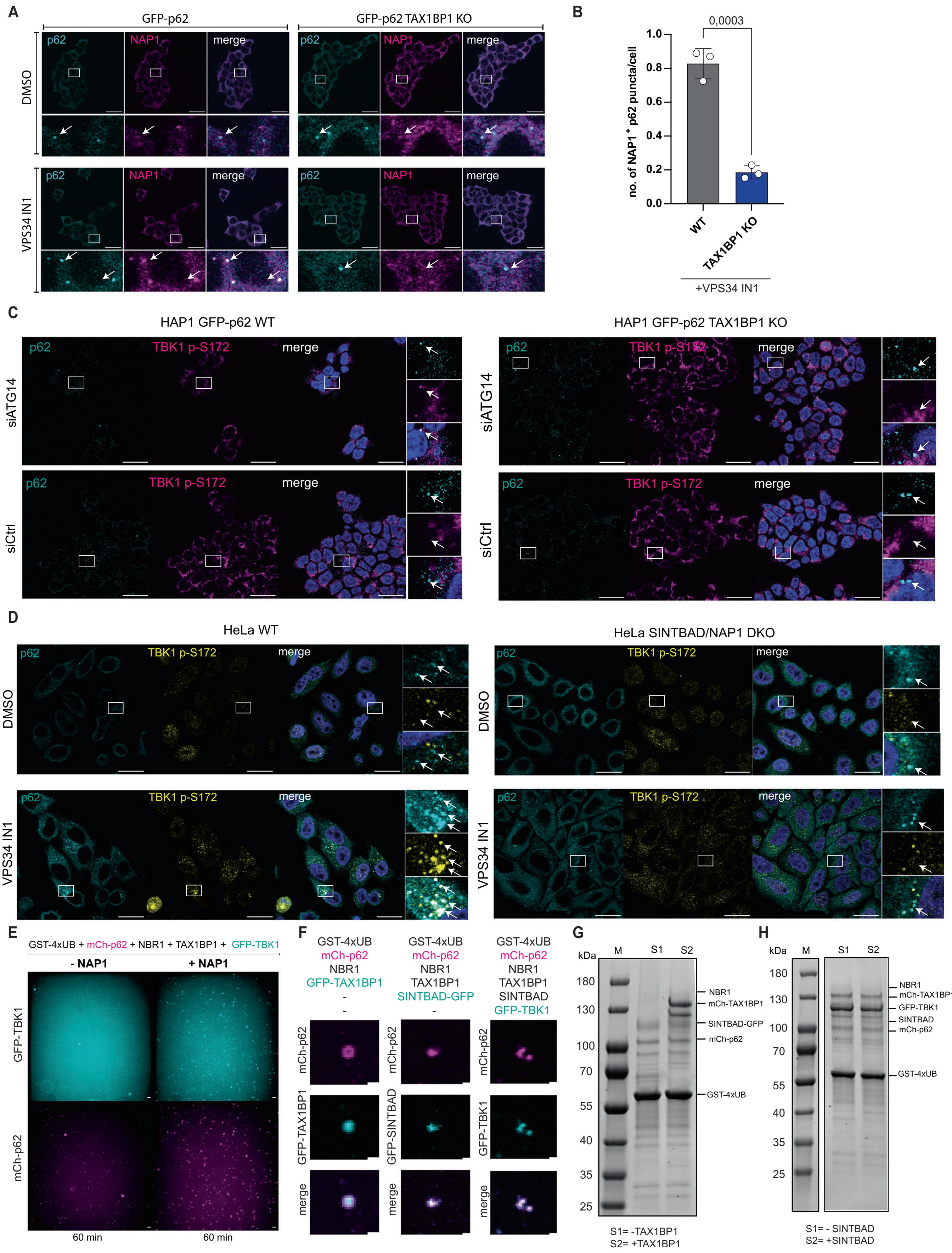
A
GFP-p62
GFP-p62 TAX1BP1 KO
p62
NAP1
merge
DMSO
VPS34 IN1
B
0,0003
no. of NAP1+ p62 puncta/cell
1.0
0.8
0.6
0.4
0.2
0.0
WT
TAX1BP1 KO
+VPS34 IN1
C
HAP1 GFP-p62 WT
HAP1 GFP-p62 TAX1BP1 KO
TBK1 p-S172
siATG14
siCtrl
D
HeLa WT
HeLa SINTBAD/NAP1 DKO
E
GST-4xUB + mCh-p62 + NBR1 + TAX1BP1 + GFP-TBK1
- NAP1
+ NAP1
GFP-TBK1
mCh-p62
60 min
F
GST-4xUB
mCh-p62
NBR1
GFP-TAX1BP1
TAX1BP1
SINTBAD-GFP
SINTBAD
GFP-TBK1
GFP-SINTBAD
G
kDa
M
S1
S2
180
130
100
70
55
40
35
25
NBR1
mCh-TAX1BP1
mCh-p62
GST-4xUB
S1= -TAX1BP1
S2= +TAX1BP1
H
S1= - SINTBAD
S2= +SINTBAD

Figure EV2. TAX1BP1 recruits TBK1 to p62 condensates via the adapter protein SINTBAD/NAP1.

(**A**) Representative immunofluorescence images of cells stained for p62 and NAP1 untreated and upon autophagy inhibition (scale bar = 20 µm). NAP1 colocalization with p62 puncta was abrogated in TAX1BP1 KO cell line (white arrows). (**B**) Quantification of the number of p62 puncta positive for NAP1 upon autophagy inhibition per cell and focal plane in (**A**). (**C**) Representative immunofluorescence images of GFP-p62 WT and TAX1BP1 KO cells stained for TBK1 p-S172 after siRNA-mediated knock-down of ATG14 (scale bar = 20 µm). The TBK1 p-S172 colocalization with p62 puncta was abrogated in TAX1BP1 KO cell line (white arrows). (**D**) Representative immunofluorescence images of HeLa WT and HeLa DKO (NAP1 & SINTBAD KO) cells stained for p62 and TBK1 p-S172 upon VPS34 IN1 treatment (scale bar = 20 µm). The TBK1 p-S172 colocalization with p62 puncta was abrogated in SINTBAD/NAP1 DKO cell line (white arrows). (**E**) Representative images of the condensation assay showing NAP1-dependent recruitment of GFP-TBK1 to p62 condensates in vitro (scale bar = 10 µm). (**F**) Representative images of p62 condensates at a higher magnification (scale bar = 1 µm). (**G**) SDS Page gel as a loading control for the condensation assay in Fig. 3G. (**H**) SDS Page gel as a loading control for the condensation assay in 3H. Data in (**B**) shows the mean +/− s.d. from three independent experiments. Unpaired *t* test was performed in (**B**). Source data are available online for this figure.

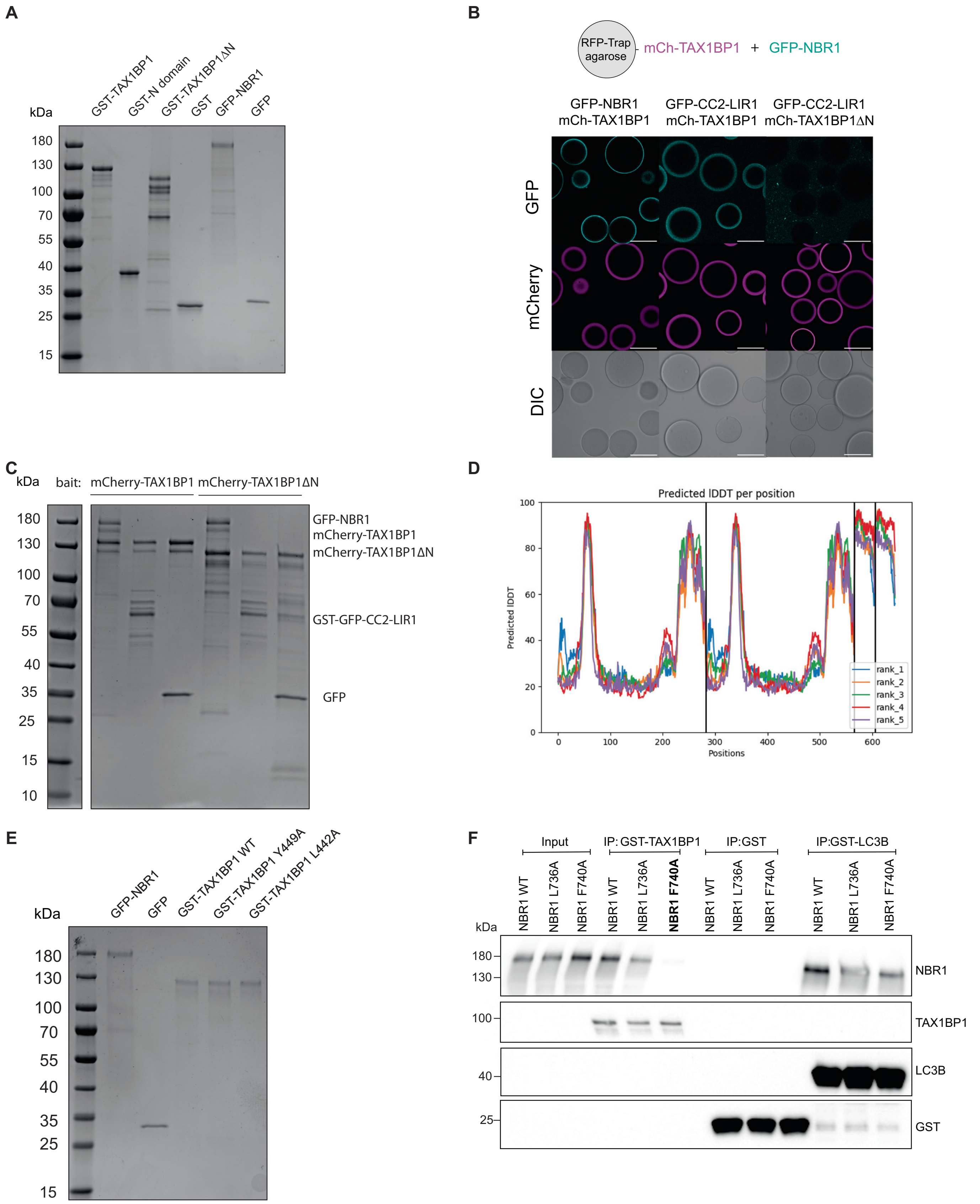
A
kDa
GST-TAX1BP1
GST-N domain
GST-TAX1BP1ΔN
GST
GFP-NBR1
GFP
180
130
100
70
55
40
35
25
15
B
RFP-Trap agarose
mCh-TAX1BP1 + GFP-NBR1
GFP-NBR1 mCh-TAX1BP1
GFP-CC2-LIR1 mCh-TAX1BP1
GFP-CC2-LIR1 mCh-TAX1BP1ΔN
GFP
mCherry
DIC
C
kDa
bait: mCherry-TAX1BP1 mCherry-TAX1BP1ΔN
180
130
100
70
55
40
35
25
15
10
GFP-NBR1
mCherry-TAX1BP1
mCherry-TAX1BP1ΔN
GST-GFP-CC2-LIR1
GFP
D
Predicted lDDT per position
Predicted lDDT
Positions
rank_1
rank_2
rank_3
rank_4
rank_5
E
kDa
GFP-NBR1
GFP
GST-TAX1BP1 WT
GST-TAX1BP1 Y449A
GST-TAX1BP1 L442A
180
130
100
70
55
40
35
25
15
F
Input
IP: GST-TAX1BP1
IP:GST
IP:GST-LC3B
NBR1 WT
NBR1 L736A
NBR1 F740A
kDa
180
130
100
40
25
NBR1
TAX1BP1
LC3B
GST

 © The Author(s)

Figure EV3. Mapping the interaction between TAX1BP1 and NBR1.

(**A**) SDS Page gel as loading control for microscopy-based interaction assay in 4 A. (**B**) Representative images for microscopy-based interaction assay with GFP tagged NBR1 fragment (CC2-LIR2) and mCherry-TAX1BP1 FL or ΔN on RFP-trap beads (scale bar = 80 µm). (**C**) SDS Page gel as a loading control for the interaction assay in (**B**). (**D**) pLDDT plot for the AlphaFold 2 prediction in Fig. 4E. (**E**) SDS Page gel as a loading control for the interaction assay in Fig. 4F. (**F**) Western blot of pulldown with cells expressing NBR1 point mutants including GST-LC3B as bait to determine the effect of point mutant on GABARAP and LC3B binding. Source data are available online for this figure.

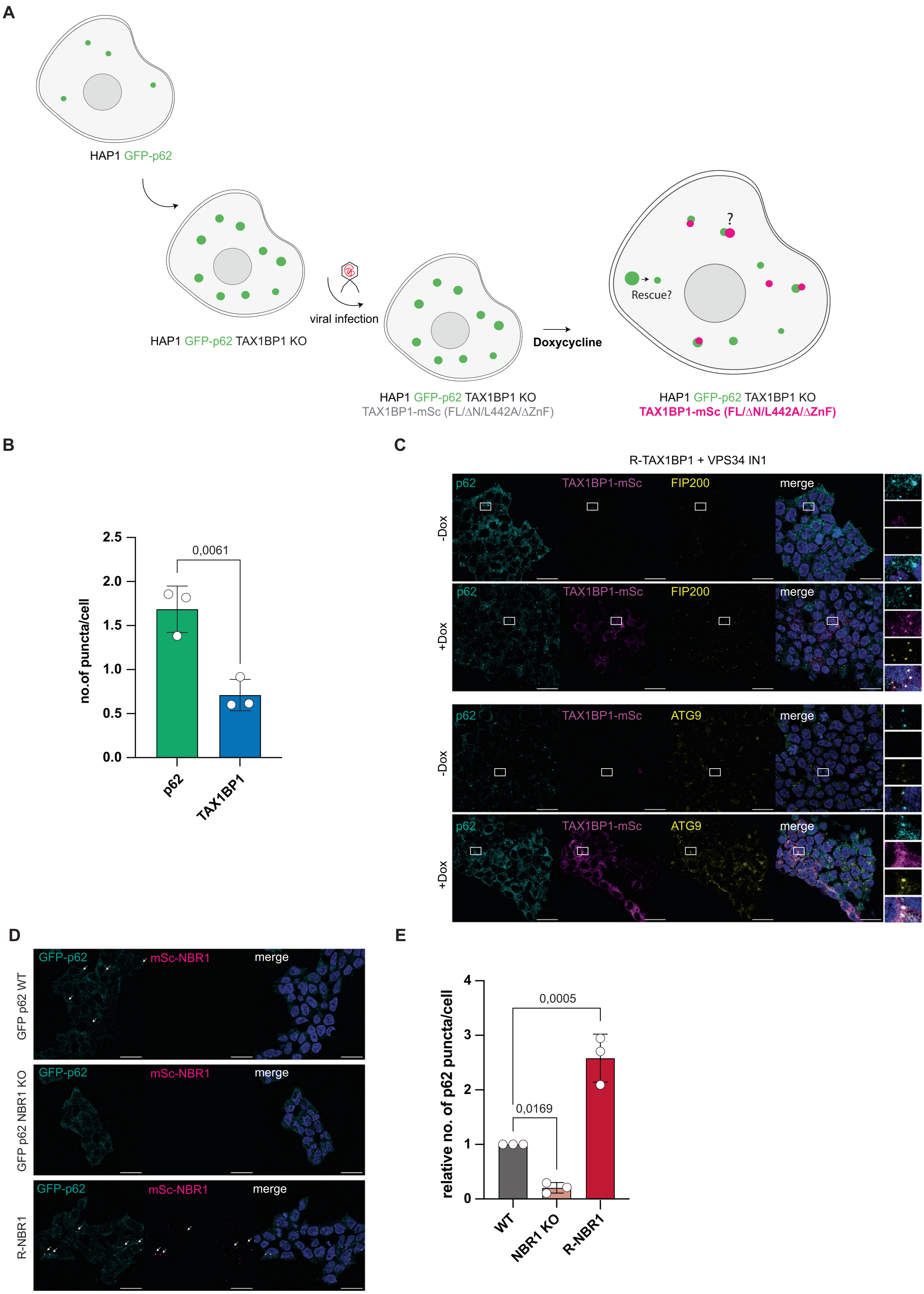
A
HAP1 GFP-p62
HAP1 GFP-p62 TAX1BP1 KO
viral infection
HAP1 GFP-p62 TAX1BP1 KO
TAX1BP1-mSc (FL/ΔN/L442A/ΔZnF)
Doxycycline
Rescue?
?
HAP1 GFP-p62 TAX1BP1 KO
TAX1BP1-mSc (FL/ΔN/L442A/ΔZnF)
B
no.of puncta/cell
0,0061
p62
TAX1BP1
C
R-TAX1BP1 + VPS34 IN1
p62
TAX1BP1-mSc
FIP200
merge
ATG9
-Dox
+Dox
D
GFP-p62
mSc-NBR1
merge
GFP p62 WT
GFP p62 NBR1 KO
R-NBR1
E
relative no. of p62 puncta/cell
0,0005
0,0169
WT
NBR1 KO
R-NBR1

 © The Author(s)

Figure EV4. The TAX1BP1–NBR1 interaction is required for the autophagic flux of p62 condensates.

(**A**) Schematic overview for the experimental set-up of the experiment shown in Fig. 6. (**B**) Quantification of the number of p62 and TAX1BP1 foci in R-TAX1BP1 cells upon VPS34 IN1 treatment per cell and focal plane. (**C**) Representative immunofluorescence images of R-TAX1BP1 cell line treated with VPS34 IN1 and +/− doxycycline to induce re-expression of TAX1BP1-mSc and stained for FIP200 and ATG9 (scale bar = 20 μm). (**D**) Representative immunofluorescence images of GFP-p62 WT, NBR1 KO and NBR1 KO cells re-expressing mSc-NBR1 (scale bar = 20 μm). p62 & mSc-NBR1 puncta are highlighted (white arrows). (**E**) Quantification of number of p62 puncta per cell and focal plane in (**D**). The number of p62 puncta was plotted as a relative to GFP-p62 WT. Data in (**B**, **E**) shows the mean +/− s.d. from three independent experiments. Unpaired *t* test was performed in (**B**). One-way ANOVA with Dunnett's multiple comparison test was performed in (**D**). Source data are available online for this figure.

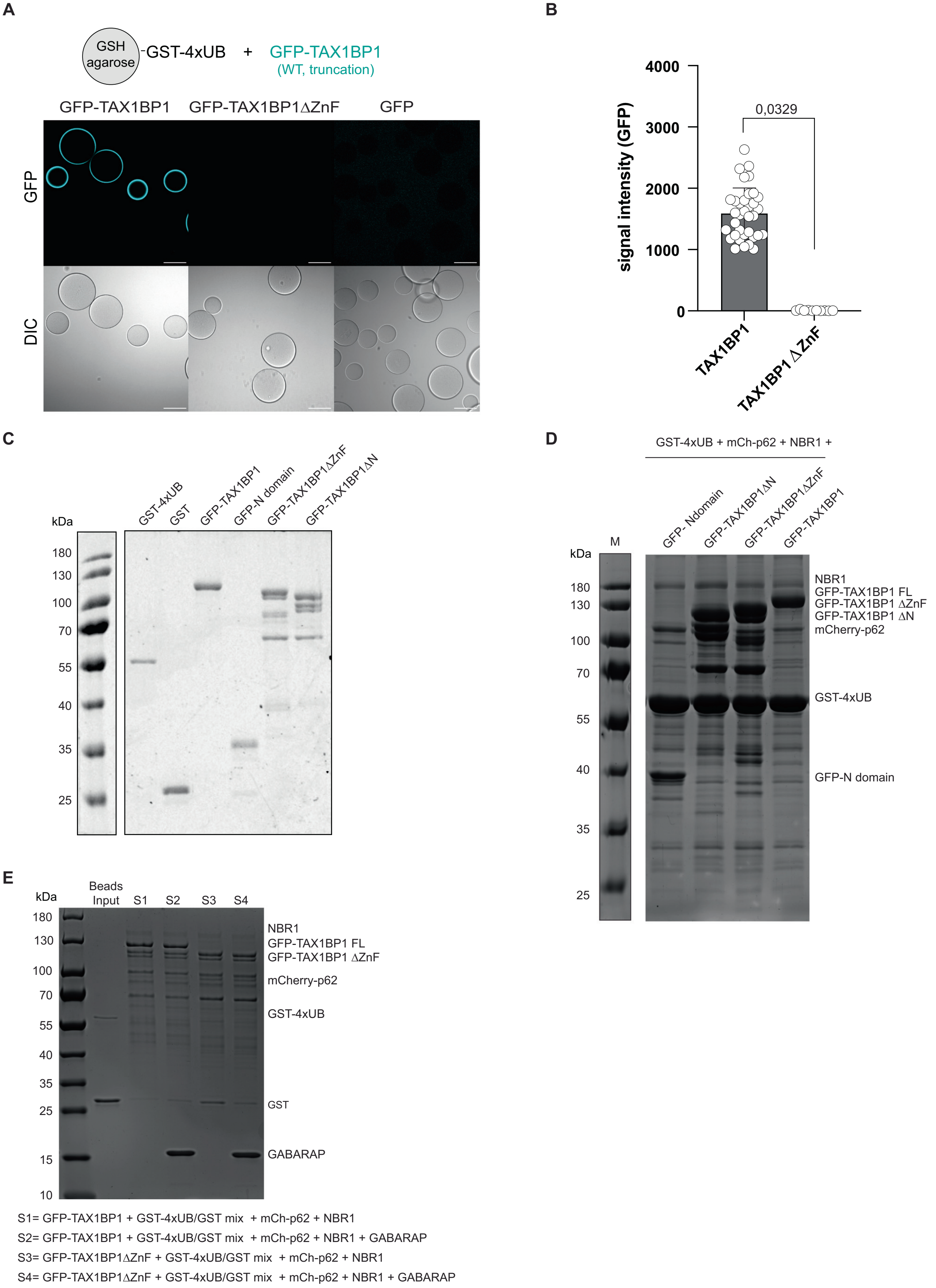
A
GSH agarose
-GST-4xUB + GFP-TAX1BP1
(WT, truncation)
GFP-TAX1BP1
GFP-TAX1BP1ΔZnF
GFP
GFP
DIC
B
signal intensity (GFP)
4000
3000
2000
1000
0
0,0329
TAX1BP1
TAX1BP1 ΔZnF
C
kDa
GST-4xUB
GST
GFP-TAX1BP1
GFP-N domain
GFP-TAX1BP1ΔZnF
GFP-TAX1BP1ΔN
180
130
100
70
55
40
35
25
D
GST-4xUB + mCh-p62 + NBR1 +
M
kDa
GFP- Ndomain
GFP-TAX1BP1ΔN
GFP-TAX1BP1ΔZnF
GFP-TAX1BP1
180
130
100
70
55
40
35
25
NBR1
GFP-TAX1BP1 FL
GFP-TAX1BP1 ΔZnF
GFP-TAX1BP1 ΔN
mCherry-p62
GST-4xUB
GFP-N domain
E
kDa
Beads Input
S1
S2
S3
S4
180
130
100
70
55
40
35
25
15
10
NBR1
GFP-TAX1BP1 FL
GFP-TAX1BP1 ΔZnF
mCherry-p62
GST-4xUB
GST
GABARAP
S1= GFP-TAX1BP1 + GST-4xUB/GST mix + mCh-p62 + NBR1
S2= GFP-TAX1BP1 + GST-4xUB/GST mix + mCh-p62 + NBR1 + GABARAP
S3= GFP-TAX1BP1ΔZnF + GST-4xUB/GST mix + mCh-p62 + NBR1
S4= GFP-TAX1BP1ΔZnF + GST-4xUB/GST mix + mCh-p62 + NBR1 + GABARAP

 © The Author(s)

Figure EV5. Ubiquitin interaction stabilizes the recruitment of TAX1BP1.

(**A**) Representative images of a microscopy-based protein–protein interaction assay using the indicated proteins (scale bar = 80 µm). (**B**) Quantification of signal intensity of GFP-TAX1BP1 and GFP-TAX1BP1ΔZnF on GST-4xUB coated beads in (**A**). (**C**) SDS Page gel as a loading control for the microscopy-based interaction assay shown in (**A**). (**D**) SDS Page gel as a loading control for the condensation assay shown in Fig. 7B. (**E**) SDS Page gel as a loading control for the microscopy-based interaction assay in Fig. 7I. The data in (**B**) shows the mean +/- s.d. from three independent experiments. Each data point in (**B**) represents the mean signal intensity for an individual bead. Background-corrected values were used for statistical analysis and negative values were excluded. Nested one-way ANOVA with Dunnett's multiple comparison test was performed in (**B**). Source data are available online for this figure.

© The Author(s)
